# New data on the longhorn beetles of Mongolia with particular emphasis on the genus *Eodorcadion* Breuning, 1947 (Coleoptera, Cerambycidae)

**DOI:** 10.3897/zookeys.739.23675

**Published:** 2018-02-22

**Authors:** Lech Karpiński, Wojciech T. Szczepański, Bazartseren Boldgiv, Marcin Walczak

**Affiliations:** 1 Department of Zoology, Faculty of Biology and Environmental Protection, University of Silesia, Bankowa 9, 40-007 Katowice, Poland; 2 Ecology Group, Department of Biology, National University of Mongolia, Ikh Surguuliin Gudamj 1, Ulaanbaatar 14201, Mongolia; 3 Academy of Natural Sciences of Drexel University, Philadelphia, PA 19103, USA

**Keywords:** *Chamaenerion angustifolium*, desert steppe, endemic species, faunistics, mountain forest steppe, Onagraceae, taiga, zoogeography

## Abstract

New data on the distribution, biology, and ecology of the longhorn beetles occurring in Mongolia are presented together with a list of 35 species that were collected during a one-month entomological expedition in August 2015. New localities of some rare taxa endemic to Mongolia, such as *Eodorcadion dorcas dorcas* (Jakovlev, 1901), *E. humerale impluviatum* (Faldermann, 1833), and *E. intermedium intermedium* (Jakovlev, 1889) are given. High-quality photographs of several rather unique species, i.e., *Pachytodes longipes* (Gebler, 1832), *Eodorcadion maurum australe* Danilevsky, 2014, *E. oryx* (Jakovlev, 1895), *Monochamus impluviatus impluviatus* (Motschulsky, 1859), and *M. sutor longulus* (Pic, 1898) along with images of their habitats or feeding grounds are also presented. Furthermore, the ecological role of the fireweed *Chamaenerion angustifolium* (L.) Scop. in the case of boreal anthophilous cerambycid species is highlighted for the first time.

## Introduction

The longhorn beetle family (Cerambycidae) is one of the most species-rich groups of beetles (Coleoptera) with approximately 35,000 described species (Švácha and Lawrence 2014). The cerambycid fauna of Mongolia is represented by 167 species (Danilevsky 2017c). Several of these species, especially ones in the genera *Brachyta* and *Eodorcadion*, are represented by a few subspecies.

Due to their almost pristine habitats, the cerambycid fauna of Mongolia is quite unique. Many boreal species, which are very rare and threatened in Europe, e.g., *Pachyta lamed* (Linnaeus, 1758), *Macroleptura thoracica* (Creutzer, 1799), *Lepturalia nigripes* (DeGeer, 1775) and *Exocentrus stierlini* Ganglbauer, 1883, are abundant in the area of the southern Siberian taiga in the north of the country. On the other hand, desert and desert steppe habitats in the southern part of the country are inhabited by many endemic longhorn beetles, especially from genera such as *Rapuzziana*, *Brachyta*, *Pachytella*, *Anoplistes*, and *Eodorcadion*.

The state of the knowledge of the longhorn fauna of Mongolia as well as information about biology and ecology of some of the species distributed in the region is still poor. Therefore, the present study aims to supplement the knowledge in this field.

## Study area and methods

Mongolia, which spans the southernmost border of the permafrost and the northernmost deserts of Inner Asia, is located in a transitional zone between the boreal forests of Siberia and the Gobi Desert. Due to its great distance from oceans, being surrounded by high mountains and being situated at a high elevation of more than one and half km above sea level on average, this landlocked country has an extreme continental climate with marked ranges of seasonal and diurnal temperatures and low amounts of precipitation. The extreme range of its temperatures varies from between -80 and 60 °C and the annual precipitation varies from 50 mm in the Gobi Desert to 400 mm in the northern mountainous area. Approximately 85% of the total precipitation falls from April to September (MNET 2009a).

The country is also characterised by a wide variety of habitats, from high mountains with taiga or forest steppe in the north, through steppes and desert steppes in the central part of the country, to the Gobi Desert in the south. The northern forests in the area of the Khan Khentey Mountains can be divided into the light taiga with *Betula platyphylla*, *Larix sibirica*, and *Pinus sylvestris* and the dark taiga with *Picea obovata*, *Abiessibirica, Pinussibirica*, and *Larix sibirica* (Ermakov et al. 2002). This wide range of relatively intact ecosystems provides suitable habitats for a variety of plant and animal species. Mongolia is one of the few countries that is still considered to be relatively untouched in regard to many environmental conditions (MNET 2009a).

**Map 1.**
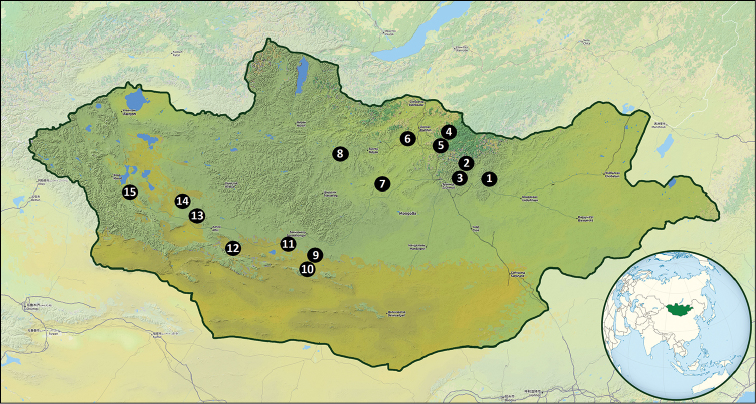
Research plots in Mongolia: **1** 40 km NE of Baganuur (47°51'N, 108°54'E) **2** four neighbouring localities: 70 km NE of Ulaanbaatar (47°57'N, 107°49'E); 75 km NE of Ulaanbaatar (48°10'N, 107°55'E); 80 km NE of Ulaanbaatar (48°06'N, 107°50'E); 80 km NE of Ulaanbaatar (48°13'N, 107°43'E) **3** 60 km E of Ulaanbaatar (47°52'N, 107°39'E) **4** four neighbouring localities: 35 km NE of Zuunkharaa (48°59'N 106°55'E); 40 km NE of Zuunkharaa (48°57'N 107°05'E); 50 km NE of Zuunkharaa (49°05'N, 107°17'E); 25 km NE of Zuunkharaa (49°04'N, 106°49'E) **5** two neighbouring localities: 10 km NE of Zuunkharaa (48°54'N, 106°43'E); 5 km E of Zuunkharaa (48°51'N, 106°36'E) **6** 5 km W of Khötöl (49°05'N, 105°29'E) **7** 20 km N of Ulaanshiveet (47°37'N, 103°51'E) **8** 5 km E of Khairkhan (48°37'N, 102°01'E) **9** 10 km S of Khairkhandulaan (45°48'N, 101°59'E) **10** five neighboring localities: 33 km S of Nariinteel (45°39'N, 101°22'E); 20 km NEE of Bogd (45°17'N, 101°02'E); 20 km SE of Bogd (45°05'N, 101°08'E); 10 km W of Baruunbayan-Ulaan (45°08'N, 101°14'E); 5 km W of Baruunbayan-Ulaan (45°10'N, 101°17'E) **11** 35 km SE of Bumbugur (45°59'N, 99°50'E) **12** two neighboring localities: 10 km NW of Biger (45°47'N, 97°02'E); 30 km NW of Biger (45°50'N, 96°45'E) **13** three neighboring localities: 20 km SSW from Bayan-Uul (46°51'N, 95°07'E); 20 km E of Sain-Ust (47°22'N, 94°42'E); 3 km E of Chuchmorit (47°21'N, 94°33'E) **14** 20 km NW of Zereg (47°23'N, 92°28'E) (OpenStreetMap contributors).

The entomological expedition, which consisted of three scientists from the Department of Zoology, University of Silesia (Poland), took place in August 2015. During the one-month-long research, several sampling trips to various locations in the northern, central, and south-western parts of Mongolia in the Töv, Selenge, Khovd, Govi-Altai, Bayankhongor, Övörkhangai, Bulgan and Khentii Aimags were carried out (Map 1). The investigations were conducted in several research plots, *inter alia* in the villages or environs of Ulaanbaatar, Erdenet, Altai, Khukhmorit, Bayankhongor, Bogd and Arvaikheer. The part of our study that focused especially on immature stages of longhorn beetles was carried out in the West Khentey region (the Khonin Nuga Research Station), which is situated in the buffer zone of the Strictly Protected Area of Khan Khentey.

The most effective standard methods for collecting beetles, such as shaking them down into an entomological umbrella, sweep netting, and analyses of the inhabited material, were used during the field research. Beetles were studied using an Optek SZM7045-J4L microscope at 7–90× magnifications. Photographs of the cerambycids in nature, their host plants and habitats, were taken with Canon EOS 550D and Canon EOS 600D cameras. Photographs of the habitus were taken with a Canon EOS 50D digital camera equipped with a MP-E 65 mm macro lens. The images that were produced were stacked, aligned, and combined using ZERENE STACKER software (www.zerenesystems.com). The geographical coordinates were read off and recorded using a Garmin Oregon 550T 3-Inch Handheld GPS Navigator. For each collected specimen, exact location (including GPS coordinates), altitude, date and names of the collectors are given. Additionally, information about general distribution and biology of the species are also provided.

The following abbreviations are used in the text:

**LK** Lech Karpiński,

**MW** Marcin Walczak,

**WTS** Wojciech T. Szczepański.

The specimens are preserved in the entomological collections of the Department of Natural History of the Upper Silesian Museum in Bytom and the Department of Biology of National University of Mongolia as well as in the authors’ private collections.

This is the second of a series of papers on longhorn beetles from the area of central-east Asia. The first one (Kadyrov et al. 2016) was devoted to Cerambycidae of west Tajikistan.

## Results

During the one-month-long expedition, a total of 36 taxa (including one subspecies) belonging to three subfamilies (Lepturinae, Cerambycinae, Lamiinae) was recorded. They represent approximately 20% of the Mongolian cerambycid fauna. The list of the recorded taxa, along with the new localities, general characteristics, and remarks on the species biology and ecology is presented here.

### Lepturinae Latreille, 1802

#### Pachyta lamed

Taxon classificationAnimaliaColeopteraCerambycidae

(Linnaeus, 1758)

Fig. 1G

##### Material examined.

Selenge Aimag: 35 km NE of Zuunkharaa, dark taiga (48°59'N, 106°55'E), 1399 m a.s.l., 05 VIII 2015, 1♂, on *Chamaenerion angustifolium*, leg. MW.

**Figure 1.**
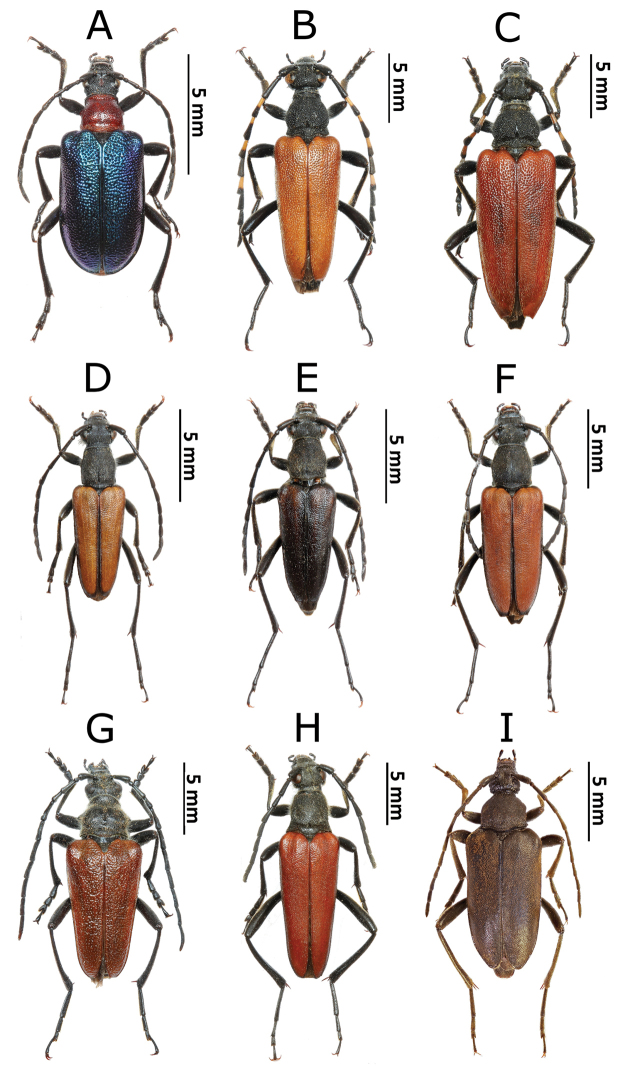
Photos of longhorn beetles specimens collected during the expedition to Mongolia in 2015: **A** *Gaurotes virginea aemula* (female) **B** *Stictoleptura variicornis* (male) **C** *S. variicornis* (female) **D** *Anastrangalia sequensi* (male) **E** *A. sequensi* (male, melanistic form) **F** *A. sequensi* (female) **G** *Pachyta lamed* (male) **H** *Lepturalia nigripes rufipennis* (male) **I** *Pachytodes longipes* (female).

##### Remarks.

*Pachyta lamed* is a widely distributed Holarctic species. In the Palaearctic region, it primarily occurs in the northern parts of Europe and Asia (Cherepanov 1990a). The species mainly inhabits coniferous forests having a large share of spruce (*Picea* spp.), which is the host plant of the larvae. The adults fly from the end of June to mid-August. After mating, the females lay eggs on the thin roots of decaying thick-trunked trees. After their third hibernation, the larvae abandon the galleries and make pupal cells in the upper layer of the soil (Cherepanov 1990a).

In Mongolia, it is rarely encountered in the taiga ecosystem (e.g., Namhaidorzh 1972, Müller et al. 2013).

Only a single male was observed on the fireweed *Chamaenerion angustifolium* on the exposed site in dark taiga habitat (Fig. 6A).

#### Pachyta quadrimaculata

Taxon classificationAnimaliaColeopteraCerambycidae

(Linnaeus, 1758)

##### Material examined.

Selenge Aimag: 40 km NE of Zuunkharaa, dark taiga (48°57'N, 107°05'E), 1270 m a.s.l., 04 VIII 2015, 2♂♂, on *Chamaenerion angustifolium*, leg. MW.

##### Remarks.

An ordinary Palaearctic species that is distributed alike *P. lamed* but in the east it only reaches the Trans-Baikal region (Cherepanov 1990a). It is definitely more frequent than the previous species. The larvae develop in the roots (under the bark and in the wood) of coniferous trees, mostly pines (*Pinus* spp.) Their biology is also similar to the previously described species (Cherepanov 1990a).

In Mongolia, *P. quadrimaculata* occurs in the taiga in the northern part of the country, although it is probably also present in Khovd Province (Namhaidorzh 1972).

We observed this species in the dark taiga habitat together with *P. lamed*.

#### Gaurotes virginea aemula

Taxon classificationAnimaliaColeopteraCerambycidae

(Mannerheim, 1852)

Fig. 1A

##### Material examined.

Selenge Aimag: 40 km NE of Zuunkharaa, dark taiga (48°57'N, 107°05'E) (Fig. 6B), 1270 m a.s.l., 04 VIII 2015, 1♀, on *Chamaenerion angustifolium*, leg. LK.

##### Remarks.

The species is distributed in the North Palaearctic region from Europe to Siberia, Sakhalin and the Korean peninsula (Sama 2002), where it is divided into five subspecies (Danilevsky 2017a). According to Danilevsky (2017), *G. virginea aemula* occurs from East Russia to the Far East, through Kazakhstan, northern Mongolia and China. It is probably polyphagous on coniferous trees. The larvae feed on dead stems, stumps and thicker branches. After two years, the larvae pupate in the soil (Cherepanov 1990a).

In Mongolia, the species is also known from Töv Aimag. It was usually observed singly, e.g., 1 ex., 18 VI 1963; 1 ex., 23–24 VII 1965 (Heyrovský 1964, 1967b). Only a single, still inactive female was found inside the calyx of *Chamaenerion angustifolium* in the morning, which may suggest that the species overnights in this plant.

#### Stictoleptura variicornis

Taxon classificationAnimaliaColeopteraCerambycidae

(Dalman, 1817)

Fig. 1B, C

##### Material examined.

Selenge Aimag: 40 km NE of Zuunkharaa, dark taiga (48°57'N, 107°05'E), 1270 m a.s.l., 04 VIII 2015, 3♂♂, 3♀♀, on *Filipendula palmata*, 7♂♂, 3♀♀, on *Chamaenerion angustifolium*, leg. MW; 10♂♂, 2♀♀, on *Filipendula palmata*, leg. WTS; 3♂♂, on *Filipendula palmata*, leg. LK; 35 km NE of Zuunkharaa (48°59'N, 106°55'E), 1399 m a.s.l., 05 VIII 2015, 3♂♂, on Apiaceae, leg. LK.

##### Remarks.

*Stictoleptura variicornis* is distributed from eastern Europe to the Pacific Ocean coast including Japan (Danilevsky 2017a). This species, which is rare in Europe, reaches the Białowieża Forest in Poland (Gutowski 1995), where it is strictly protected by European law. It inhabits dead thick-trunked trees and the stumps of various coniferous and deciduous species. The larva initially lives under the bark and then in the wood, where it usually makes a pupal cell after its second hibernation. The flight period of this beetle occurs between the second half of June to mid-August (Cherepanov 1990a). It is a common species in the Mongolian taiga. It was also recorded from Bulgan Aimag by Heyrovský (1973a).

Although the specimens were observed on the flowers of a few plant species, they seemed to prefer *Filipendula palmata* (Fig. 6C).

#### Anastrangalia sequensi

Taxon classificationAnimaliaColeopteraCerambycidae

(Reitter, 1898)

Figs 1D–F , 6D

##### Material examined.

Töv Aimag: 70 km NE of Ulaanbaatar (47°57'N, 107°49'E), 1833 m a.s.l., 30 VII 2015, 2♂♂, on *Seseli condensatum*, leg. MW; 5♂♂ (2♂ melanistic form), on *Seseli condensatum*, leg. WTS; 1♀, on *Seseli condensatum*, leg. LK; 80 km NE of Ulaanbaatar (48°06'N, 107°50'E) (Fig. 6E), 1538 m a.s.l., 31 VII 2015,1♀, on *Apiaceae*, leg. LK; 4♂♂, 2♀♀, on flowers near river, leg. MW; 80 km NE of Ulaanbaatar (48°13'N, 107°43'E), 1778 m a.s.l., 31 VII 2015, 1♂, on *Apiaceae*, leg. LK; Selenge Aimag: 40 km NE of Zuunkharaa (48°57'N, 107°05'E), 1270 m a.s.l., 04 VIII 2015, 3♀♀, on *Chamaenerion angustifolium*, leg. WTS; 35 km NE of Zuunkharaa (48°59'N, 106°55'E), 1399 m a.s.l., 05 VIII 2015, 1♂, 1♀, on *Apiaceae*, leg. WTS; 1♂, on *Apiaceae*, leg. LK; Khentii Aimag: 40 km NE of Baganuur (47°51'N, 108°54'E), 1612 m a.s.l., 22 VIII 2015, 1♂, leg. WTS.

##### Remarks.

This species is only distributed in northern Asia, where its range is from the Urals to the Pacific Ocean including Japan and in the south, it reaches northern Mongolia and China (Cherepanov 1990a). Its flight period lasts from the end of May to the third week of August. After mating, the females lay eggs in the bark crevices of the stumps and trunks of various standing coniferous trees. The life cycle of this species usually lasts two years with pupation in spring and early summer (Cherepanov 1990a).

Although it is one of the most common anthophilous species in Mongolia, it is distributed exclusively in the northern part of the country. The species is rather variable regarding the colour of the elytra, which ranges from brownish yellow to black. Entirely black specimens of *A. sequensi* might be confused with *Anastrangalia renardi* (Gebler, 1848), which differs mainly in its more parallel-sided elytra and their outer angles that are rounded.

#### Lepturobosca virens

Taxon classificationAnimaliaColeopteraCerambycidae

(Linnaeus, 1758)

##### Material examined.

Selenge Aimag: 40 km NE of Zuunkharaa, dark taiga (48°57'N, 107°05'E), 1270 m a.s.l., 04 VIII 2015, 4♂♂, 3♀♀, on *Chamaenerion angustifolium*, leg. MW; 05 VIII 2015, 1♀ on *Chamaenerion angustifolium*, leg. LK.

##### Remarks.

This ordinary Palaearctic species is distributed from the Atlantic to the Pacific Ocean (Danilevsky 2017a), where it mainly inhabits mountain and taiga zones. *Lepturobosca virens* is ecologically related with coniferous trees (*Pinus*, *Picea*, *Abies*) but it is also known from *Betula* (Danilevskaya et al. 2009). The species is very abundant locally (e.g., Siberia) (Cherepanov 1990a).

#### Pachytodes longipes

Taxon classificationAnimaliaColeopteraCerambycidae

(Gebler, 1832)

Fig. 1I

##### Material examined.

Selenge Aimag: 50 km NE of Zuunkharaa (49°05'N, 107°17'E), 930 m a.s.l., 02 VIII 2015, 1♀, leg. MW.

##### Remarks.

The species is distributed in northern Asia eastwards from about Baikal Lake to the Pacific Ocean. According to Danilevsky (2014a), all records for Altay and Tuva were connected with misidentifications of *Pachytodes bottcheri* (Pic, 1911). The southern edge of its occurrence reaches the forest areas of northern Mongolia, China, and Korea (Cherepanov 1990a, Danilevsky 2017a). *Pachytodes longipes* is probably a polyphagous species, which usually inhabits the basal zones of deciduous wood species. The larvae develop in rotten wood around and in roots. Adults were found from July to August. The species is characterised by the highly variable colour of the elytra (Cherepanov 1990a).

The species was recorded in Mongolia by Lobanov et al. (1981), but with no specific locality data (Danilevsky 2017b). It is well known from Töv and Arkhangai Aimags (Danilevsky 2014a). Recently, one specimen of this species was also collected by Müller et al. (2013) in the West Khentey region.

A single specimen was caught flying in the light taiga habitat next to a river (Fig. 6F). Our finding confirms its presence in this region.

#### Oedecnema gebleri

Taxon classificationAnimaliaColeopteraCerambycidae

(Ganglbauer, 1889)

##### Material examined.

Khentii Aimag: 40 km NE of Baganuur (47°51'N, 108°54'E), 1612 m a.s.l., 22 VIII 2015, 4 larvae, *Larix sibirica*, leg. LK, WTS.

##### Remarks.

*Oedecnema gebleri* is distributed from Eastern Europe (Ukraine and Russia) to the Pacific Ocean (Danilevsky 2017a). Its polyphagous larvae develop in the basal zones of dead trees and in the stumps of both deciduous and coniferous species. Pupation occurs in the wood or in the soil. The life cycle usually lasts two or three years. The adults feed on various flowers from the end of May to August (Švácha and Danilevsky 1989, Cherepanov 1990a).

The larvae (Fig. 6G) were found at ground level in the basal zones of thin larches *Larix sibirica* in the forest steppe (Fig. 6H). It is a rather common species in the Mongolian taiga and forest steppe.

#### Macroleptura thoracica

Taxon classificationAnimaliaColeopteraCerambycidae

(Creutzer, 1799)

##### Material examined.

Selenge Aimag: 25 km NE of Zuunkharaa (49°04'N, 106°49'E), 1399 m a.s.l., 01 VIII 2015, 1♂, 1♀, dead imagines, *Populus tremula*, leg. LK.

##### Remarks.

The species is distributed from northern and eastern Europe through Siberia, northern China, Mongolia, the Korean peninsula, and Sakhalin to Japan (Danilevsky 2017a). In Europe, this species is strictly protected by European law. Although *Macroleptura thoracica* is mostly polyphagous on deciduous trees (Švácha and Danilevsky 1989, Sama 2002), it was also observed on the fir *Abies* sp. in Japan (Sama 2002). The species inhabits the dead, rotten wood of thick trunks. The adults fly from June to August and usually stay on their host plants; they rarely visit flowers (Sama 2002). In Mongolia, this is rare species that is not numerous (e.g., Namhaidorzh 1972, 1976a).

The remains of two specimens were found deep in the wood of the trunk of the stately poplar *Populus tremula* together with numerous larval feeding grounds. This primeval forest relict species, which is very rare in Europe, appears to be rather numerous in this region. This indicates the high degree of the naturalness of the Mongolian habitats.

#### Leptura aethiops

Taxon classificationAnimaliaColeopteraCerambycidae

Poda von Neuhaus, 1761

##### Material examined.

Selenge Aimag: 40 km NE of Zuunkharaa (48°57'N, 107°05'E), 1270 m a.s.l., 04 VIII 2015, 1♂, on *Chamaenerion angustifolium*, leg. MW.

##### Remarks.

*Leptura aethiops* occurs in almost the entire Palaearctic region from France to Japan (Sama 2002, Danilevsky 2017a). The species has a broad spectrum of host plants. The larvae usually develop in the stumps of dead deciduous trees but they have occasionally been found in conifers (Švácha and Danilevsky 1989, Sama 2002).

#### Leptura annularis

Taxon classificationAnimaliaColeopteraCerambycidae

Fabricius, 1801

##### Material examined.

Selenge Aimag: 40 km NE of Zuunkharaa (48°57'N, 107°05'E), 1270 m a.s.l., 04 VIII 2015, 1♂, on *Filipendula palmata*, leg. MW; 2♂♂, 2♀♀, on *Chamaenerion angustifolium*, leg. MW; 4♂♂, 1♀, on *Chamaenerion angustifolium*, leg. WTS; 2♂♂, on *Chamaenerion angustifolium*, leg. LK; 35 km NE of Zuunkharaa (48°59'N, 106°55'E), 1399 m a.s.l., 05 VIII 2015, 1♀, on *Chamaenerion angustifolium*, leg. LK.

##### Remarks.

This species is widely distributed from Europe, where it is considered to be a montane subalpine species, to the Pacific Ocean, including Mongolia, China, and the Korean peninsula. A closely related species *Leptura mimica* Bates, 1884 is endemic to Japan and Sakhalin Islands. Its divergence has been confirmed in both morphological and genetic studies (Makihara and Saito 1985, Makihara et al. 1991, Saito et al. 2002, Rossa et al. 2017). The biology of *L. annularis* is similar to that of the previously described species (Švácha and Danilevsky 1989).

#### Lepturalia nigripes rufipennis

Taxon classificationAnimaliaColeopteraCerambycidae

(Blessig, 1873)

Fig. 1H

##### Material examined.

Töv Aimag: 70 km NE of Ulaanbaatar (47°57'N, 107°49'E), 1833 m a.s.l., 30 VII 2015, 1♂, on *Seseli condensatum*, leg. MW; Selenge Aimag: 50 km NE of Zuunkharaa (49°05'N, 107°17'E), 930 m a.s.l., 02 VIII 2015, 8 larvae, *Betula platyphylla*, leg. LK, MW, and WTS.

##### Remarks.

This is a temperate Palaearctic species that is distributed from north-eastern Europe to the Far East (Švácha and Danilevsky 1989, Sama 2002). Moreover, the subspecies *L. nigripes rufipennis* is known to occur in the easternmost part of its territory from East Siberia and Kazakhstan to the Far East (Danilevsky 2017a).

In Mongolia, it is a quite common species, especially in stands having a large share of birch trees (e.g., Namhaidorzh 1972, Cherepanov 1990a, Müller et al. 2013), which is the main preferred host plant species. The development of the larvae takes place in trunks, branches, and decaying stumps. The adults fly from May to August and feed on the flowers of various species (Cherepanov 1990a, Sama 2002).

A single male was observed on flowers in the forest steppe (Fig. 7A). The larvae (Fig. 7B) were found in the rotten stumps of the birch *Betula platyphylla* in their basal zones in the light taiga (Fig. 7C). Like *L. thoracica*, this species, which is very rare in Europe, seems to be rather common in pristine local habitats of both Mongolian taiga and forest steppe.

#### Stenurella melanura

Taxon classificationAnimaliaColeopteraCerambycidae

(Linnaeus, 1758)

##### Material examined.

Selenge Aimag: 40 km NE of Zuunkharaa (48°57'N, 107°05'E), 1270 m a.s.l., 04 VIII 2015, 3♂♂, 2♀♀, on *Achillea asiatica*, leg. MW; 1♂, on *Chamaenerion angustifolium*, leg. MW; 1♀, on *Achillea asiatica*, leg. WTS.

##### Remarks.

This is a species that is very common and widespread throughout Europe and the Palaearctic part of Asia (Danilevsky 2017a). It is a polyphagous species, whose larvae feed on the decaying wood of stumps or branches of both deciduous and coniferous trees (Sama 2002).

### Cerambycinae Latreille, 1802

#### Clytus arietoides

Taxon classificationAnimaliaColeopteraCerambycidae

Reitter, 1900

Fig. 2A

##### Material examined.

Töv Aimag: 75 km NE of Ulaanbaatar (48°10'N, 107°55'E), 1589 m a.s.l., 30 VII 2015 (22 II 2016, ex cult), 1♀, from *Larix sibirica*, leg. MW.

**Figure 2.**
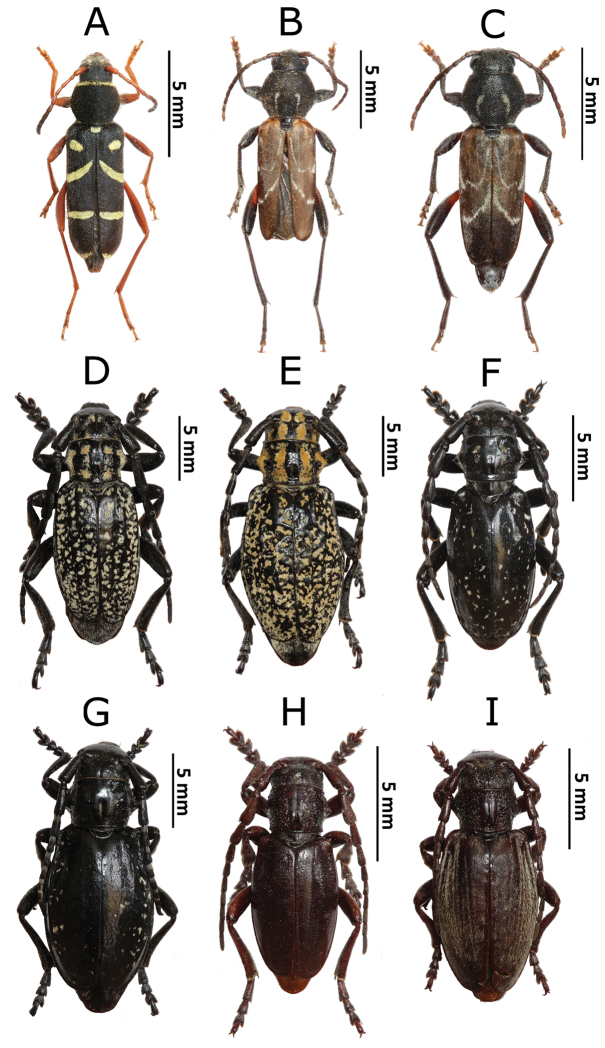
Photos of longhorn beetles specimens collected during the expedition to Mongolia in 2015: **A** *Clytus arietoides* (female) **B** *Xylotrechus hircus* (male) **C** *X. hircus* (female) **D** *Eodorcadion humerale impluviatum* (male) **E** *E. humerale impluviatum* (female) **F** *E. humerale impluviatum* (male, Khentey Mountains) **G** *E. humerale impluviatum* (female, Khentey Mountains) **H** *Eodorcadion carinatum involvens* (male) **I** *E. carinatum involvens* (female).

##### Remarks.

This oriental species is distributed widely from the Urals to Sakhalin and Japan (Sama 2002, Danilevsky 2017a). It is ecologically associated with coniferous forests. The larvae develop in dead or drying trunks and twigs of various conifers, especially larches. After two years, the larvae pupate in wood during summer and the imagines emerge the next spring. The adults fly from May to August and, during the mating season, they stay on their host plants and occasionally visit flowers (Švácha and Danilevsky 1988, Cherepanov 1990b).

In Mongolia, the species is also known, *inter alia*, from Khovd Aimag (Heyrovský 1969).

One female was reared from a branch of a fallen larch *Larix sibirica* collected in the forest steppe (Fig. 7D). The same material was inhabited by larvae of *Monochamus impluviatus* (Motschulsky, 1859) and *M. sutor* (Linnaeus, 1758).

#### Xylotrechus hircus

Taxon classificationAnimaliaColeopteraCerambycidae

(Gebler, 1825)

Fig. 2B, C

##### Material examined.

Selenge Aimag: 50 km NE of Zuunkharaa (49°05'N, 107°17'E), 930 m a.s.l., 02 VIII 2015 (24 III 2016, ex cult), 1♂, from *Betula platyphylla*, leg. MW; (09 III 2016, ex cult), 1♀, from *Betula platyphylla*, leg. WTS.

##### Remarks.

*Xylotrechus hircus* occurs exclusively in Northern Asia from Altai to Japan (Cherepanov 1990b, Danilevsky 2017a). The species is ecologically associated with birch, which is the only known host plant to date. The larvae initially live under the bark and then in the wood where they pupate after about two years of development. The imagines are usually active from June to July (Cherepanov 1990b).

This is a rather infrequent taxon in Mongolia and is absent in most of the papers about this region. Recently, two specimens of this species were collected in the West Khentey region (Müller et al. 2013).

One male (Fig. 2B) and one female (Fig. 2C) were reared from the top part of a broken trunk of the birch *Betula platyphylla* (approx. 10 cm in diameter) (Fig. 7E) found in the light taiga habitat (Fig. 7F). The same material was additionally inhabited by larvae of *Aegomorphus obscurior* (Pic, 1904), *Saperda scalaris* (Linnaeus, 1758) and *Mesosa myops* (Dalman, 1817).

#### Xylotrechus pantherinus

Taxon classificationAnimaliaColeopteraCerambycidae

(Savenius, 1825)

##### Material examined.

Selenge Aimag: 50 km NE of Zuunkharaa (49°05'N, 107°17'E), 930 m a.s.l., 02 VIII 2015, 1 larva, *Salix* sp., leg. LK.

##### Remarks.

This species is widespread in the Palaearctic region and is distributed from western Europe to the Far East (Danilevsky 2017a). It is monophagous on *Salix* (*S. caprea* in Europe and *S. fragilis*, *S. sibirica* and *S. xerophila* in Siberia). The larvae develop in healthy or weakened trunks and branches, where they feed deep in the wood. Adults can be found on their host plants from June to August (Cherepanov 1990b, Sama 2002).

A single larva (Fig. 7G) was found deep in the wood of a standing willow trunk *Salix* sp. (Fig. 7H) in the light taiga next to a river.

#### Amarysius altajensis

Taxon classificationAnimaliaColeopteraCerambycidae

(Laxmann, 1770)

##### Material examined.

Selenge Aimag: 50 km NE of Zuunkharaa (49°05'N, 107°17'E), 930 m a.s.l., 02 VIII 2015, 20 larvae, *Malus baccata* and *Prunus padus*, leg. LK, MW and WTS; 40 km NE of Zuunkharaa (48°57'N, 107°05'E), 1270 m a.s.l., 04 VIII 2015, several larvae, (09 II 2016, ex cult), 1♂, from *Salix* sp., leg. WTS.

##### Remarks.

This species is distributed from Kazakhstan to the Far East, including northern Mongolia, China, and the Korean peninsula (Danilevsky 2017a). This forest species inhabits small diameter shoots and twigs of various deciduous plant species. Its life cycle lasts about three years. The imagines fly from May to June and frequently visit flowers (Švácha and Danilevsky 1988, Cherepanov 1990b).

It seems to be a rather numerous taxon in the Mongolian taiga. Several dozen larvae (Fig. 8A) were frequently found in shoots and twigs of three deciduous plant species, *Malus baccata* (Fig. 8B), *Prunus padus* (Fig. 8C) and *Salix* sp. (Fig. 8D), in both light and dark taiga. The larvae which were found in *Malus* wood were definitely more yellowish (Fig. 8E). In this area, fifteen specimens of *A. altajensis* were collected by Müller et al. (2013).

#### Anoplistes halodendri minutus

Taxon classificationAnimaliaColeopteraCerambycidae

Hammarström, 1892

##### Material examined.

Govi-Altai Aimag: 20 km E of Sain-Ust (47°22'N, 94°42'E), 1646 m a.s.l., 12–13 VIII 2015, several larvae, *Caragana bungei*, leg. LK, MW; Övörkhangai Aimag: 10 km W of Baruunbayan-Ulaan (45°08'N, 101°14'E), 1264 m a.s.l., 18 VIII 2015, several larvae and remains of one imago, *Caragana leucophloea*, leg. LK, MW.

##### Remarks.

*Anoplistes halodendri* is an east-Palaearctic species that is distributed from the Balkans to the Russian Far East, China, Korea and Japan (Danilevskaya et al. 2009). Within its range, it was divided into seven subspecies (Danilevsky 2017a): *A. h. balcanicus* Sláma, 2010, *A. h. ephippium* (Steven & Dalman, 1817), *A. h. halodendri* (Pallas, 1773), *A. h. heptapotamicus* (Semenov, 1926), *A. h. kasatkini* Lazarev, 2014, *A. h. minutus* Hammarström, 1892 and *A. h. pirus* (Arakawa, 1932). The species is ecologically associated with deciduous trees and shrubs (e.g., *Acacia*, *Daphne mezereum*, *Quercus*) in the steppe and forest-steppe habitats. Adults begin emerging in July (Cherepanov 1990b).

The larvae (Fig. 8F, G) collected in the first locality (Fig. 8H) in stems of *Caragana bungei* (Fig. 9A) clearly belong to the genus *Anoplistes*, but the exact species could not be identified with certainty. They are preliminarily classified in this taxon since it is the most common *Anoplistes* species in the country. Moreover, *minutus* is the only subspecies of *A. halodendri* that occurs in Mongolia. It was already recorded from this Province by Heyrovský (1969) and additionally from Khovd and Ömnögovi Aimags (Heyrovský 1965, 1969). It is worth noting that one of the larvae among this material belongs to the tribe Clytini, most probably to the genus *Chlorophorus*.

Regarding the second locality (Fig. 9B), several larvae were found together with the remains of a single male imago in stems of *Caragana leucophloea*. In both cases, the larval feeding grounds (Fig. 9C) were located from a few centimetres below to approx. 10 cm above ground level. Most of the emergence holes (Fig. 9D) of the adults were found on stems approx. a few centimetres above ground level. Both research plots are located in semi-desert habitats.

Apart from *Anoplistes halodendri minutus*, four other species of this genus were already recorded for Mongolia: *A. gobiensis* Namkhaidorzh, 1973, *A. kozlovi* Semenov & Znoiko, 1934, *A. mongolicus mongolicus* Ganglbauer, 1889 and *A. tuvensis* Tsherepanov, 1978 (Danilevsky 2017a). *Anoplistes tuvensis* is distributed exclusively in the region of the Tuva basin and it is ecologically associated with *Nanophyton erinaceum* (Cherepanov 1990b). *Anoplistes kozlovi* was recorded, *inter alia*, from Dundgovi, Ömnögovi and Govi-Altai Aimags (Heyrovský 1965, 1968) and *A. mongolicus* from Khovd, Govi-Altai, Ömnögovi, Bayankhongor and Dundgovi Aimags (Heyrovský 1968, 1970). However, taxonomy, distribution (especially in the Mongolia and China region) and biology of most of the species in this genus need to be thoroughly studied and revised.

### Lamiinae Latreille, 1825

#### Aegomorphus obscurior

Taxon classificationAnimaliaColeopteraCerambycidae

(Pic, 1904)

Fig. 5A

##### Material examined.

Selenge Aimag: 50 km NE of Zuunkharaa (49°05'N, 107°17'E), 930 m a.s.l., 02 VIII 2015 (10 IX 2016, ex cult), 1♂, from *Betula platyphylla*, leg. WTS.

##### Remarks.

*Aegomorphus obscurior* was recently raised to the species level by Hilszczański (2008). After the revision of specimens, it is known to be broadly distributed in Russia and in the Siberian part of Kazakhstan (Danilevsky and Shapovalov 2007) as well as in Mongolia (Hilszczański 2008). In Europe, it reaches Latvia (Telnov 2016) and eastern Poland (Hilszczański 2008, Danilevsky 2017a). The species was recently included in the Mongolian fauna (Hilszczański 2008) based on four specimens that were collected 30 km north of Batsumber by B. Burakowski and H. Szelęgiewicz in 1963. The larvae feed on dead trees or dead parts of living trees of their host plants: *Quercus robur* (Hilszczański and Bystrowski 2005), *Betula* sp. and *Alnus* sp. (Danilevsky and Shapovalov 2007). The life cycle lasts two years. The adults are active from the second half of May (Hilszczański and Bystrowski 2005).

A single male was reared from the top part of a broken trunk of the birch *Betula platyphylla* (approx. 10 cm in diameter) (Fig. 7E) that was found in the light taiga habitat (Fig. 7F). The same material was additionally inhabited by larvae of *Xylotrechus hircus*, *Saperda scalaris*, and *Mesosa myops*. Our findings constitute the second record of this species from Mongolia.

#### Saperda similis

Taxon classificationAnimaliaColeopteraCerambycidae

Laicharting, 1784

##### Material examined.

Selenge Aimag: 50 km NE of Zuunkharaa (49°05'N, 107°17'E), 930 m a.s.l., 03 VIII 2015, 1 larva, *Salix* sp., leg. LK.

##### Remarks.

*Saperda similis* is a rather rare but widespread species that is distributed from Europe to the Far East (Danilevsky 2017a). Although this species is ecologically associated with willows (Cherepanov 1991b), according to Sama (2002), it is probably monophagous on *Salix caprea*. The larvae develop in thin stems and branches of willows that are still growing (Cherepanov 1991b). The adults are active at dusk and during the night in June and July and can be found on their host plants (Sama 2002).

A single larva in a pupal cell (Fig. 9E) was found in the trunk of a recently dead willow *Salix* sp. in the light taiga next to a river (Fig. 9F).

#### Saperda scalaris hieroglyphica

Taxon classificationAnimaliaColeopteraCerambycidae

(Pallas, 1773)

##### Material examined.

Selenge Aimag: 50 km NE of Zuunkharaa (49°05'N, 107°17'E), 930 m a.s.l., 02 VIII 2015, 2 larvae, *Betula platyphylla*, leg. LK.

##### Remarks.

*Saperda scalaris hieroglyphica* is distributed from European Russia to the Far East (Danilevsky 2017a). This subspecies is sometimes recognised as a synonym of the nominotypical subspecies (e.g., Sama 2002) from which it differs only by its whitish not yellowish pubescence. In Asia, this polyphagous species is ecologically more associated with birch (Cherepanov 1991b).

Two larvae (Fig. 9G), which were boring in a thick layer of cambium in a broken trunk of *Betula platyphylla* (of diameter approx. 20 cm), were found under the bark (Fig. 9H) in the light taiga habitat (Fig. 7F). The same material was additionally inhabited by *Xylotrechus hircus*, *Aegomorphus obscurior* and *Mesosa myops* in their immature stages.

#### Saperda alberti

Taxon classificationAnimaliaColeopteraCerambycidae

Plavilstshikov, 1916

##### Material examined.

Selenge Aimag: 50 km NE of Zuunkharaa (49°05'N, 107°17'E), 930 m a.s.l., 03 VIII 2015, 1 larva, *Salix* sp., leg. LK.

##### Remarks.

This East-Asian species is distributed from western Siberia throughout north Kazakhstan, Mongolia, and China as well as to the Far East and Japan (Danilevsky 2017a). The larvae develop under bark that has recently died and in the wood of certain deciduous plants, e.g., *Populus*, *Salix*, *Chosenia*. They pupate in sapwood or under or inside the bark. The imagines are active from early June to mid-August and can be found on their host plants or they are sometimes attracted to artificial light sources (Cherepanov 1991b, Danilevskaya et al. 2009).

A single early larval instar was found under the bark of a broken trunk of willow *Salix* sp. in the light taiga. A large number of this species was collected in this area by Müller et al. (2013).

#### Agapanthia pilicornis pilicornis

Taxon classificationAnimaliaColeopteraCerambycidae

(Fabricius, 1787)

Fig. 5H

##### Material examined.

Selenge Aimag: 50 km NE of Zuunkharaa (49°05'N, 107°17'E), 930 m a.s.l., 02 VIII 2015, 2♂♂, leg. MW.

##### Remarks.

*Agapanthia pilicornis pilicornis* is distributed in the Ussuri-Primor’e region, Trans-Baikal, Sakhalin, northern Mongolia, northeast China, Korean peninsula, and Japan (Cherepanov 1991a). The second subspecies, *A. pilicornis laushanensis* Breuning, 1965, is known exclusively from two Chinese provinces: Henan and Shāndōng (Danilevsky 2017a). Little is known about the biology of this species. The imagines are active in June and July, but the life cycle and the preimaginal stages are not clearly understood (Cherepanov 1991a). This species is quite similar to *Agapanthia amurensis* Kraatz, 1879; however, it can be easily distinguished *inter alia* by variegated antennae and darker body colour.

This is a rather infrequent taxon in Mongolia and is absent in most of the papers about this region (e.g., Heyrovský 1964–1975). The only records of approx. ten specimens, collected from the second half of June to mid-July, are included in the work of Namhaidorzh (1972).

Our finding extends the known period of occurrence of this species to the beginning of August. Two males were collected in the light taiga habitat (Fig. 7C) by sweep-netting method.

#### Eodorcadion carinatum involvens

Taxon classificationAnimaliaColeopteraCerambycidae

(Fischer von Waldheim, 1823)

Figs 2H, I , 10A–C

##### Material examined.

Töv Aimag: 60 km E of Ulaanbaatar (47°52'N, 107°39'E), 1499 m a.s.l., 30 VII 2015, 29♂♂, 13♀♀ (5♀♀ white pubescence form (Fig. 10C)), leg. MW; 20♂♂, 8♀♀ (2♀♀ white pubescence form), leg. WTS; 11♂♂, 10♀♀ (4♀♀ white pubescence form), leg. LK; 80 km NE of Ulaanbaatar (48°13'N, 107°43'E), 1778 m a.s.l., 31 VII 2015, 3♂♂, 2♀♀, leg. MW; 1♂, 1♀, leg. WTS; 1♂, leg. LK; Selenge Aimag: 10 km NE of Zuunkharaa (48°54'N, 106°43'E), 999 m a.s.l., 05 VIII 2015, 1♂, 1♀, leg. LK; 2♂♂, leg. MW; 2♂♂, 2♀♀, leg. WTS; 5 km E of Zuunkharaa (48°51'N, 106°36'E), 916 m a.s.l., 05 VIII 2015, 1♂, 1♀, leg. WTS; 5 km W of Khötöl [Хөтөл] (49°05'N, 105°29'E), 809 m a.s.l., 06 VIII 2015, 1♂, 1♀, leg. LK; 1♂, leg. WTS; Arkhangai Aimag: 15 km S of Khairkhan (48°22'N, 101°52'E), 1437 m a.s.l., 07 VIII 2015, 1♂, leg. LK; 5 km E of Khairkhan (48°37'N, 102°01'E), 1398 m a.s.l., 07 VIII 2015, 1♀ (white pubescence form), leg. WTS.

##### Remarks.

*Eodorcadion carinatum involvens* is one of the five subspecies that have already been described; they are distributed from the Jenisei River to the Far East. This taxon is the most common and widespread in the northern and central parts of Mongolia where it has been recorded from many localities; to the south, it reaches the Mongolian and Gobi Altai Mountains. The imagines are active from the end of June to August (Namhaidorzh 1972, Danilevsky 2007).

We observed a mass occurrence of this species (more than one hundred specimens) approx. 60 km E of Ulaanbaatar during moderately warm (22 °C) and cloudy weather conditions in the steppe habitat (Fig. 10D) at the turn of July and August. Most of the specimens were found in the interstices of the grass where they were mating. Single specimens were walking slowly. Some specimens were also found at a higher elevation (1778 m a.s.l) on xerothermic mountain slopes (Fig. 10E) sympatrically with *Eodorcadion humerale impluviatum* (Faldermann, 1833) and *Monochamus impluviatus impluviatus* (Motschulsky, 1859). Another plot, which was located approx. 15 km S of Khairkhan (48°22'N, 101°52'E), turned out to be the most westward locality of this taxon towards the city of Altai. Towards the Khangai Mountains, we did not find any further individuals despite checking numerous plots.

#### Eodorcadion maurum australe

Taxon classificationAnimaliaColeopteraCerambycidae

Danilevsky, 2014

**Figure 3.**
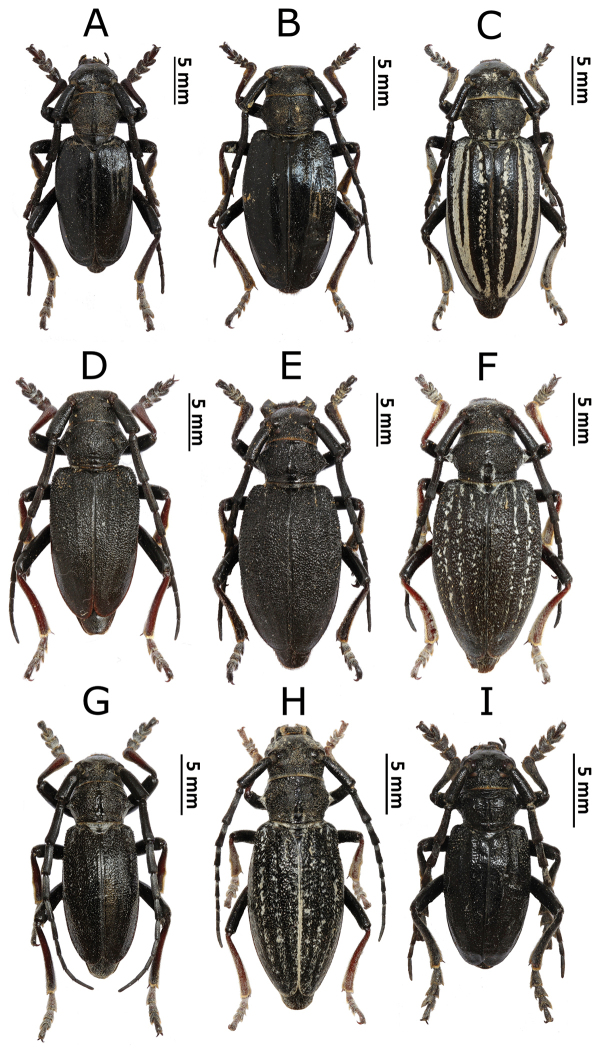
Photos of longhorn beetles specimens collected during the expedition to Mongolia in 2015: **A** *Eodorcadion consentaneum* (male) **B** *E. consentaneum* (female, black form) **C** *E. consentaneum* (female, striped form) **D** *Eodorcadion dorcas scabrosum* (male) **E** *E. dorcas scabrosum* (female, black form) **F** *E. dorcas scabrosum* (female, striped form) **G** *Eodorcadion dorcas dorcas* (male) **H** *E. dorcas dorcas* (female) **I** *Eodorcadion maurum australe* (male).

**Figure 4.**
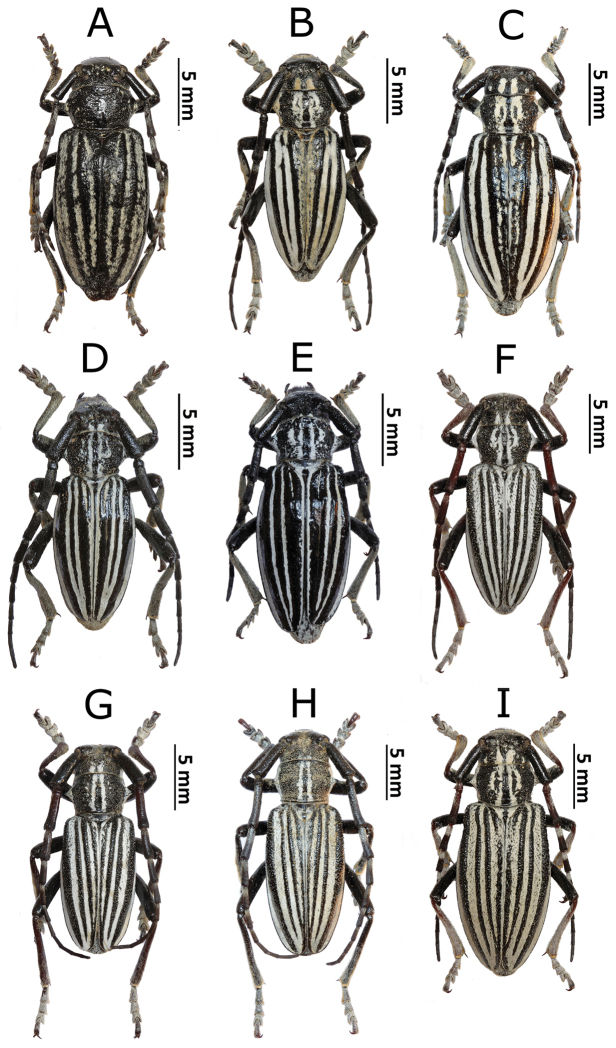
Photos of longhorn beetles specimens collected during the expedition to Mongolia in 2015: **A** *Eodorcadion maurum australe* (female) **B** *Eodorcadion oryx* (male) **C** *E. oryx* (female) **D** *Eodorcadion exaratum argali* (male) **E** *E. exaratum argali* (female) **F** *Eodorcadion intermedium intermedium* (male, reddish form) **G** *E. intermedium intermedium* (male, intermediate form) **H** *E. intermedium intermedium* (male, blackish form) **I** *E. intermedium intermedium* (female, reddish form).

Figs 3I , 4A , 10F, G

##### Material examined.

Khovd Aimag: 20 km NW of Zereg (47°23'N, 92°28'E), 1158 m a.s.l., 14 VIII 2015, 10♂♂, 2♀ (1♀ dead – remains), leg. LK; 21♂♂, leg. MW; 14♂♂, leg. WTS.

##### Remarks.

This is a recently described subspecies that is distributed in the northern and central parts of Khovd Aimag. All previously known specimens were collected from end of June to July (Danilevsky 2014b). Two other taxa occur in Mongolia: *E. m. katharinae* Reitter, 1898 and *E. m. maurum* Jakovlev, 1889 (Danilevsky 2017c).

Before noon, during rather windy and cold weather, nearly 50 individuals were observed on the border of tall and short grass meadows (Fig. 10H). The population was dominated by males (Fig. 10F); the only living female (Fig. 10G) was collected in the afternoon at the end of our stay at this locality. The males behaved rather apathetically, hiding and still drying from the morning dew in the grass. The females are likely to have been more active later than males when the weather conditions had improved. The remains of approx. ten males and one female were also found in the grass.

**Figure 5.**
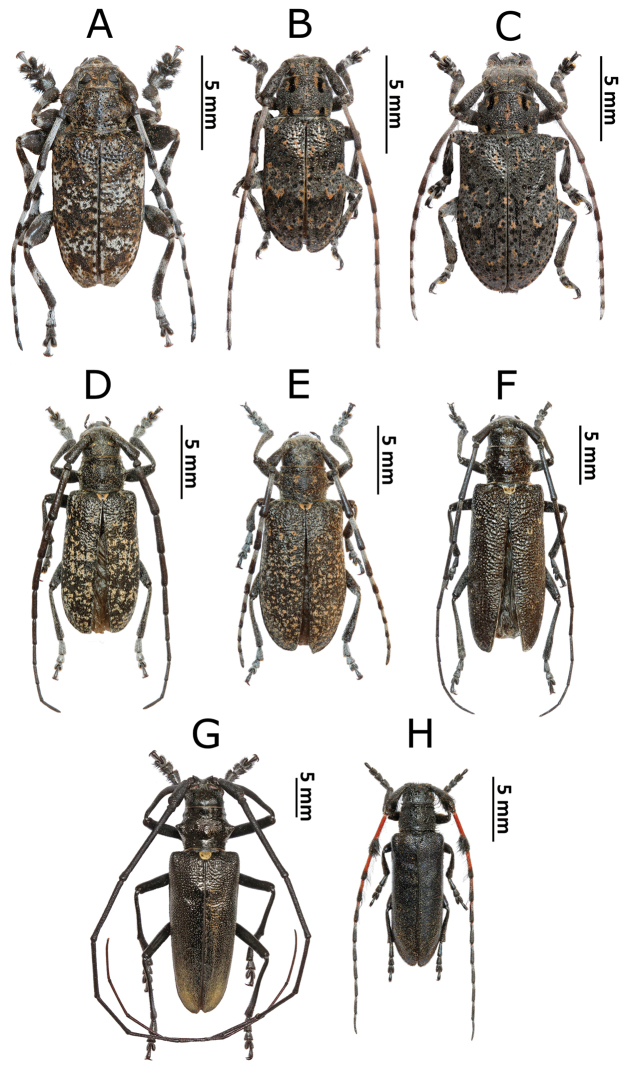
Photos of longhorn beetles specimens collected during the expedition to Mongolia in 2015: **A** *Aegomorphus obscurior* (male) **B** *Mesosa myops* (male) **C** *M. myops* (female) **D** *Monochamus impluviatus impluviatus* (male) **E** *M. impluviatus impluviatus* (female) **F** *Monochamus sutor longulus* (female) **G** *Monochamus sartor urussovii* (male) **H** *Agapanthia pilicornis pilicornis* (male).

**Figure 6.**
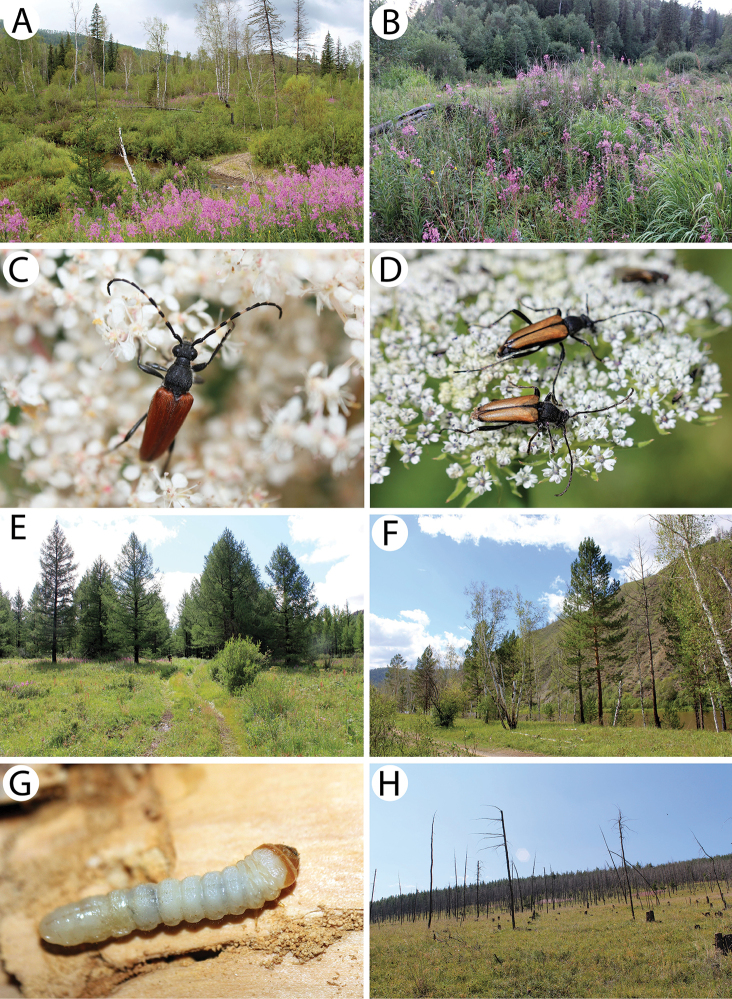
Field photos of imagines in nature, immature stages and habitats of typical Mongolian cerambycid species: **A** spruces, firs and birches in dark taiga, the habitat of *Pachyta lamed*, *P. quadrimaculata* and *Lepturobosca virens* **B** site with fireweed *Chamaenerion angustifolium* in dark taiga, the habitat of several anthopilous species e.g., *Gaurotes virginea aemula*, *Stictoleptura variicornis* **C** male of *S. variicornis* on *Filipendula palmata* **D** males of *Anastrangalia sequensi* on *Seseli condensatum* **E** larches in forest steppe, the habitat of *A. sequensi* **F** riverbank in light taiga, the habitat of *Pachytodes longipes* **G** larva of *Oedecnema gebleri* in larch wood **H** burned larches in forest steppe, the habitat of *O. gebleri* and *A. sequensi*.

**Figure 7.**
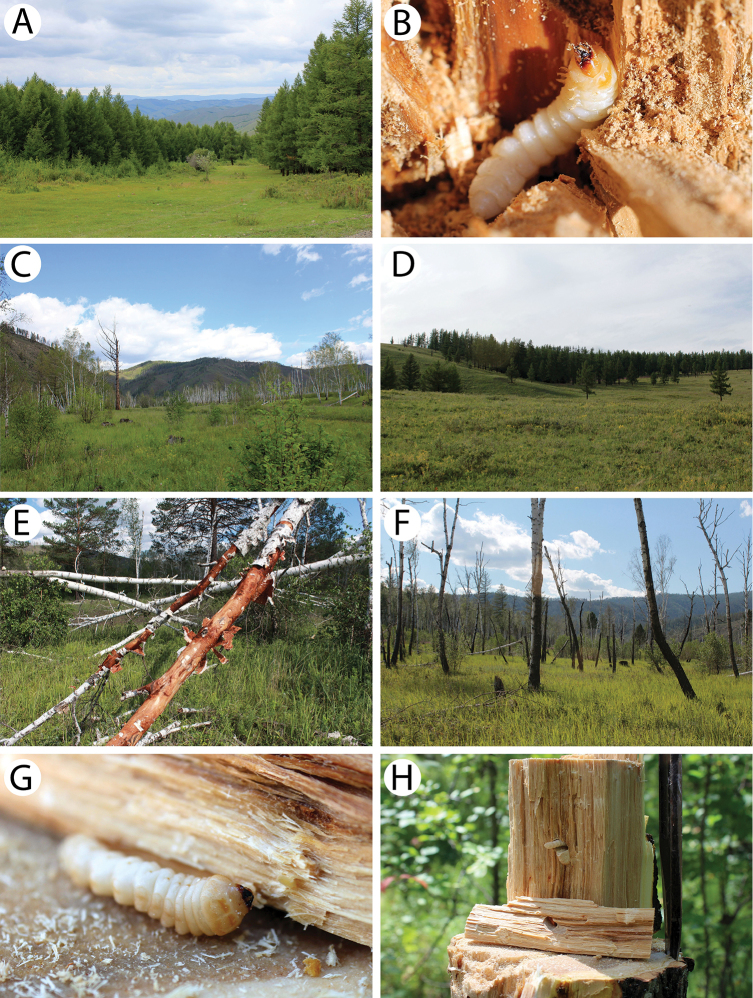
Field photos of immature stages and habitats of typical Mongolian cerambycid species: **A** larches in forest steppe, the habitat of *Lepturalia nigripes rufipennis* **B** larva of *L. nigripes* in a birch stump **C** site in light taiga, the habitat of *L. nigripes rufipennis* and *Agapanthia pilicornis pilicornis* **D** site in forest steppe, the habitat of *Clytus arietoides*, *Monochamus impluviatus impluviatus* and *M. sutor longulus* **E** broken trunk of the birch in light taiga, the microhabitat of *inter alia Xylotrechus hircus* and *Aegomorphus obscurior* **F** birches in light taiga, the habitat of *X. hircus*, *A. obscurior*, *Saperda scalaris hieroglyphica* and *Mesosa myops* **G** larva of *Xylotrechus pantherinus* **H** larva of *X. pantherinus* deep in the wood of a standing willow trunk.

**Figure 8.**
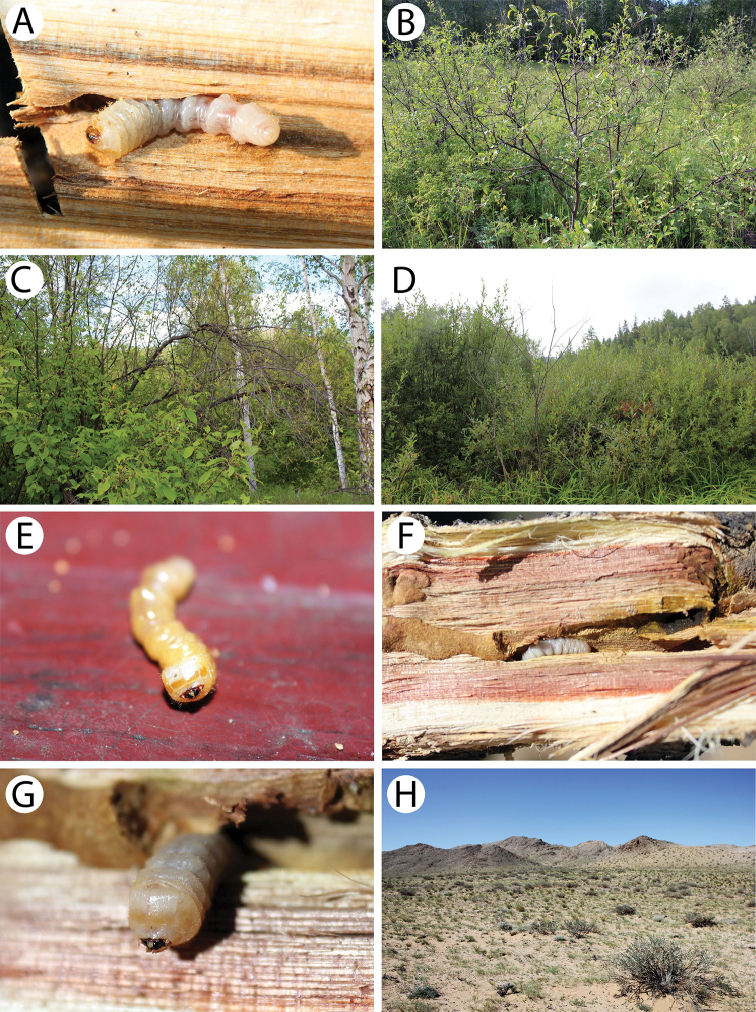
Field photos of immature stages and habitats of typical Mongolian cerambycid species: **A** larva of *Amarysius altajensis* in a bird cherry branch **B** site with young Siberian crab apple trees in light taiga, the habitat of *A. altajensis* **C** dead branches of bird cherry in light taiga, the microhabitat of *A. altajensis* **D** willow bushes in dark taiga, the habitat of *A. altajensis* **E** yellowish larva of *A. altajensis* found in a Siberian crab apple branch **F** *Anoplistes* larva in its feeding ground in a stem of *Caragana bungei* **G** larva of *Anoplistes* from *C. bungei* (detailed view) **H** *C. bungei* shrubs in semi-desert, the habitat of *Anoplistes* sp.

**Figure 9.**
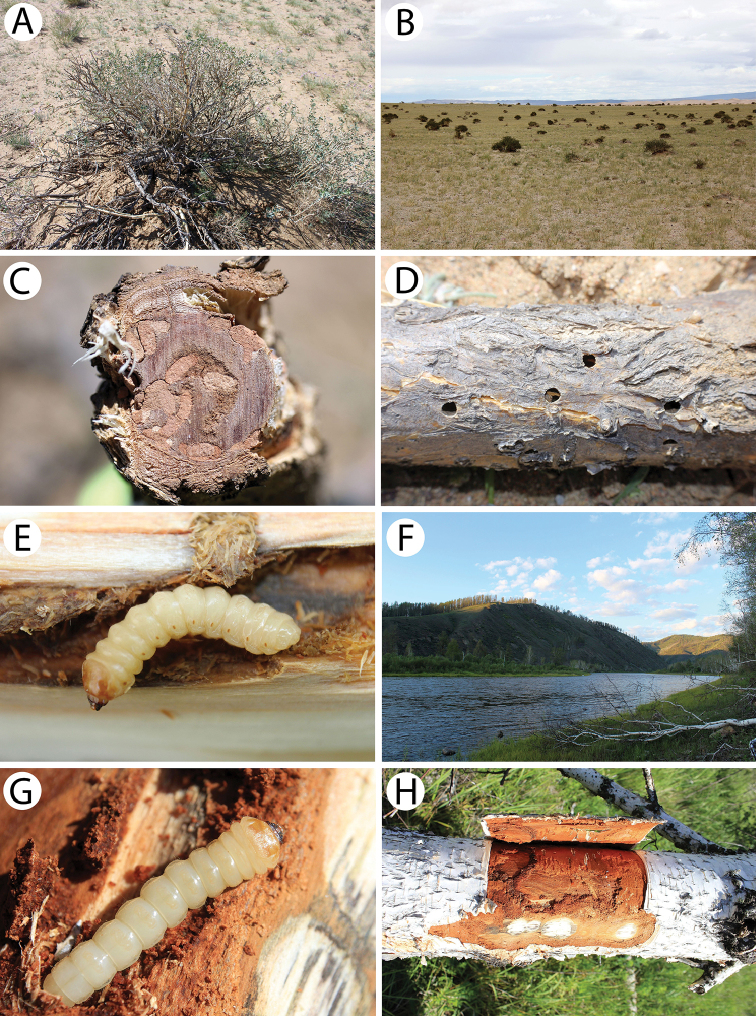
Field photos of immature stages and habitats of typical Mongolian cerambycid species: **A** shrub of *Caragana bungei*, the host plant of *Anoplistes* and *Chlorophorus* species **B** *Caragana leucophloea* shrubs in semi-desert, the habitat of *Anoplistes halodendri minutus* **C** cross-section of larval feeding grounds of *Anoplistes halodendri* in *Caragana* stem **D** adults emergence holes of *Anoplistes halodendri* **E** larva of *Saperda similis* in its pupal cell **F** riverbank in light taiga, the habitat of *S. similis* **G** larva of *Saperda scalaris* **H** thick layer of cambium under the bark of broken birch trunk, the microhabitat of *S. scalaris* and *Mesosa myops*.

**Figure 10.**
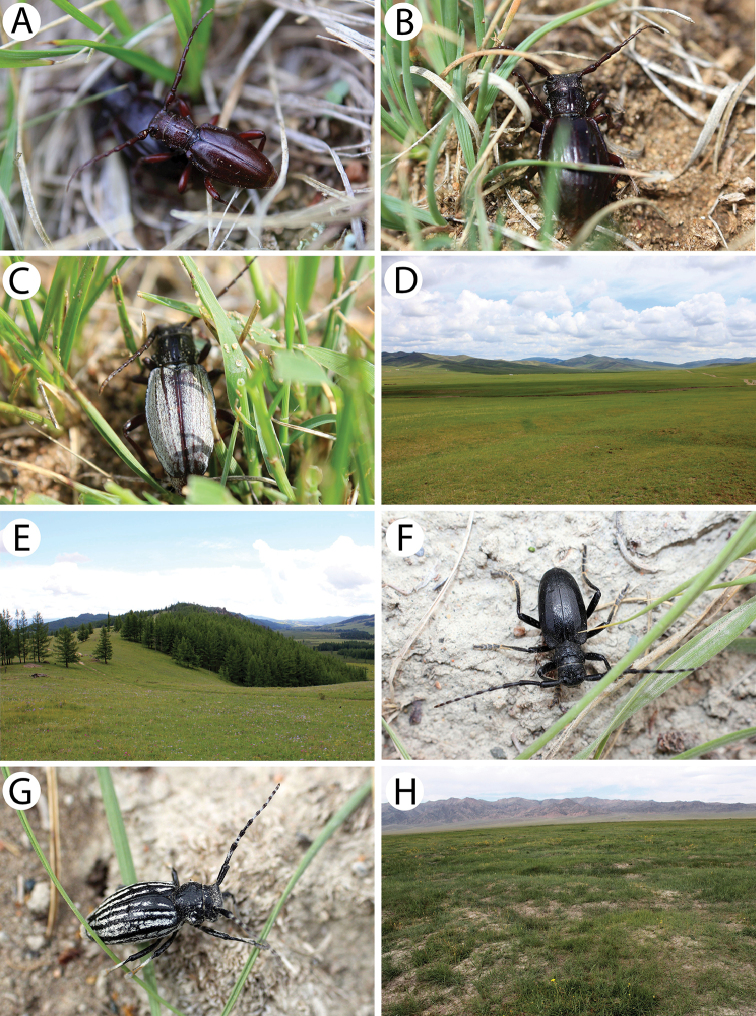
Field photos of imagines in nature and habitats of typical Mongolian cerambycid species: **A** male of *Eodorcadion carinatum involvens* **B** female of *E. carinatum involvens* **C** female of *E. carinatum involvens* (white pubescence form) **D** steppe in Ulaanbaatar environs, the habitat of *E. carinatum involvens* **E** xerothermic mountain slopes, the habitat of *E. carinatum involvens* and *Eodorcadion humerale impluviatum* **F** male of *Eodorcadion maurum australe* **G** female of *E. maurum australe* **H** short grass meadow in Zereg environs, the habitat of *E. maurum australe*.

#### Eodorcadion dorcas dorcas

Taxon classificationAnimaliaColeopteraCerambycidae

(Jakovlev, 1901)

Figs 3G, H , 11A

##### Material examined.

Govi-Altai Aimag: 20 km SSW of Bayan-Uul (46°51'N, 95°07'E), 1878 m a.s.l., 11 VIII 2015, 1♂, 2♀♀, leg. MW; 4♂♂, leg. LK.

##### Remarks.

This taxon is endemic to Mongolia and is distributed from the Khovd environs to the west and the southern parts Zavkhan Aimag and then to the northern part of Gobi-Altai Aimag (Danilevsky 2007).

**Figure 11.**
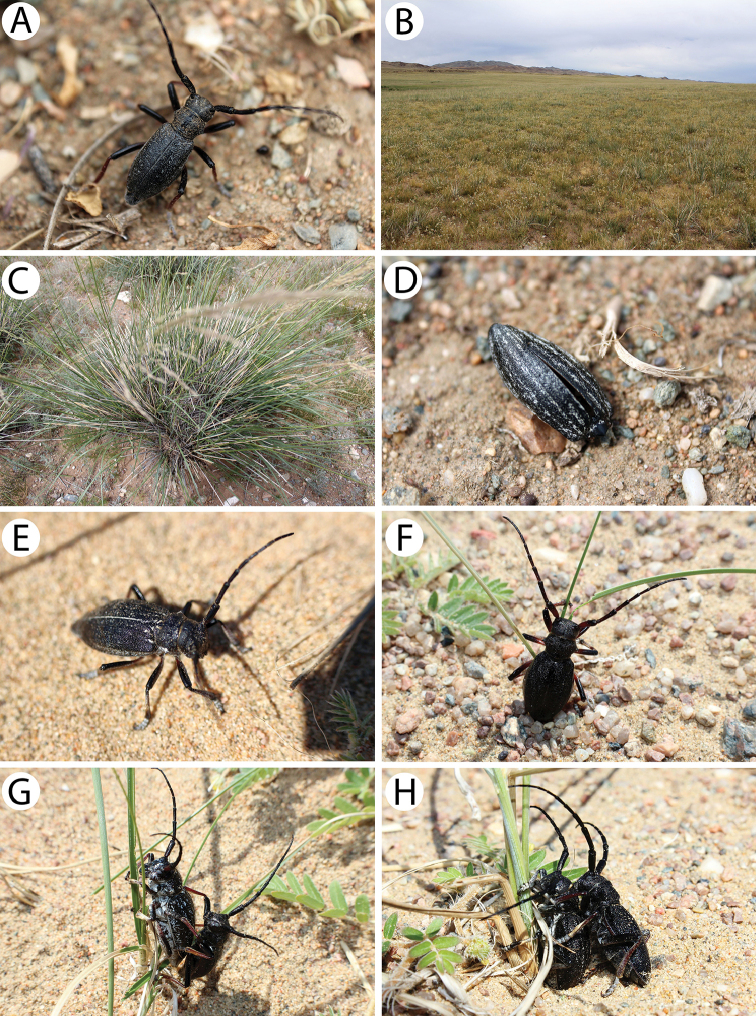
Field photos of imagines in nature and habitats of typical Mongolian cerambycid species: **A** male of *Eodorcadion dorcas dorcas* **B** small enclave with high tufts of grass, the habitat of *E. dorcas dorcas* **C** tuft of needlegrass *Achnatherum splendens*, the possible host plant of larvae of *E. dorcas dorcas* **D** remains of a female of *E. dorcas dorcas* **E** male of *Eodorcadion dorcas scabrosum* **F** female of *E. dorcas scabrosum* during eating a blade of *Iris tenuifolia* **G** pair of *E. dorcas scabrosum in copula* on *I. tenuifolia* **H** attempt to mate while laying eggs by a female of *E. dorcas scabrosum* in the roots of *I. tenuifolia*.

Only a few specimens were collected in the late afternoon in a small enclave (Fig. 11B) with high tufts of grass *Achnatherum splendens* (Fig. 11C) during rather cold (22 °C), cloudy and windy weather. The males (Fig. 11A) were rather mobile, running and trying to hide in the tufts of grass. In this locality, the remains of several imagines were found (Fig. 11D).

#### Eodorcadion dorcas scabrosum

Taxon classificationAnimaliaColeopteraCerambycidae

Namkhaidorzh, 1972

Figs 3D–F , 11E–H

##### Material examined.

Govi-Altai Aimag: 3 km E of Khukhmorit [Хөхморьт] (47°21'N, 94°33'E), 1470 m a.s.l., 13 VIII 2015, 31♂♂, 19♀♀ (12♀♀ striped form, 7♀♀ black form), leg. MW; 29♂♂, 8♀♀ (3♀♀ striped form, 5♀♀ black form), leg. WTS; 19♂♂, 8♀♀ (4♀♀ striped form, 4♀♀ black form), leg. LK.

##### Remarks.

The taxon is endemic to Mongolia; all hitherto known specimens were collected in the Khukhmorit environs (Danilevsky 2007).

**Figure 12.**
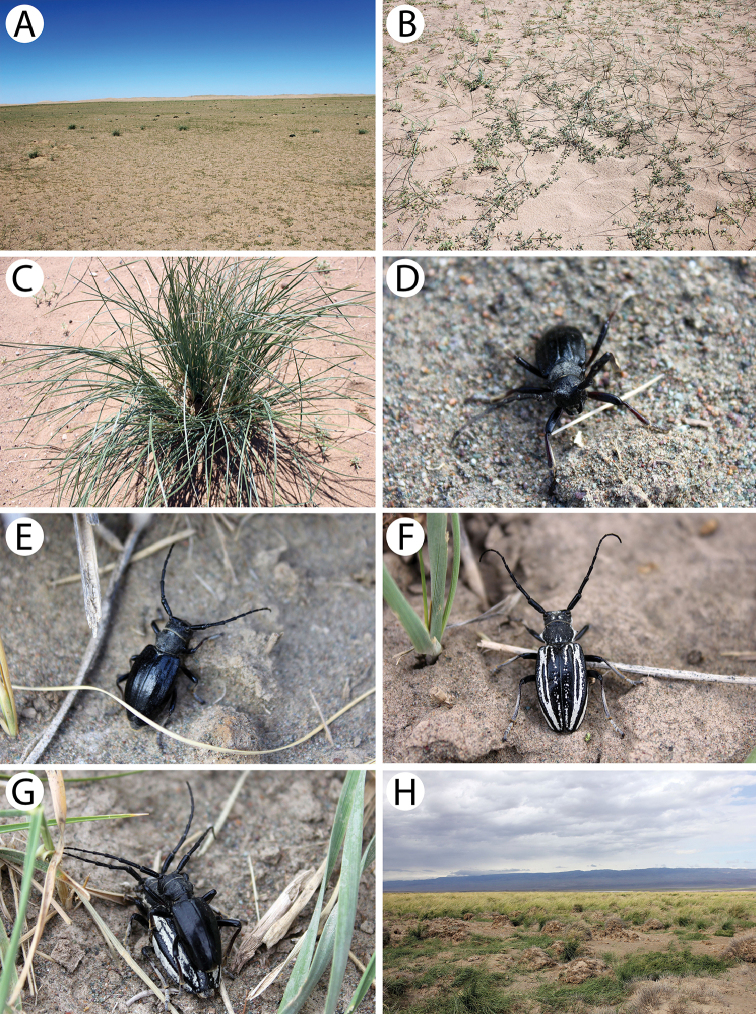
Field photos of imagines in nature and habitats of typical Mongolian cerambycid species: **A** semi-desert in Khukhmorit environs, the habitat of *Eodorcadion dorcas scabrosum* **B** detailed view of the habitat of *E. dorcas scabrosum* **C** tuft of perennial *Iris tenuifolia*, the most likely host plant of larvae of *E. dorcas scabrosum* **D** male of *Eodorcadion consentaneum* **E** female of *E. consentaneum* (black form) **F** female of *E. consentaneum* (striped form) **G** pair of *E. consentaneum in copula* **H** periphery of a drying lake with a sandy substrate and tufts of high grass, the habitat of *E. consentaneum*.

All of the specimens were collected from one plot in a semi-desert habitat (Fig. 12A, B) with very poor vegetation. The most common plant species on the plot was *Iris tenuifolia* (Fig. 12C), and therefore, it can be possibly the host plant of the larvae. Moreover, we have observed the females eating the leaf blades of irises (Fig. 11F, G) as well as apparently laying the eggs in their roots (Fig. 11F, H). During a hot (25 °C) and sunny evening, we observed plenty of incredibly active individuals (more than two hundred), whose males (Fig. 11E) were quickly moving on the sand. The females were mainly hiding in the tufts of irises and moved only occasionally. The population was dominated by males (ratio of approx. 3:1) and most of the observed females were copulating (Fig. 11G, H). This was probably the climax of the appearance of this species. It is worth noting that we have never observed any of the Dorcadionini species in such a barren desert habitat.

#### Eodorcadion consentaneum

Taxon classificationAnimaliaColeopteraCerambycidae

(Jakovlev, 1899)

Figs 3A–C , 12D–G

##### Material examined.

Govi-Altai Aimag: 10 km NW of Biger [Бигэр] (45°47'N, 97°02'E), 1331 m a.s.l., 15 VIII 2015, 29♂♂, 35♀♀ (18♀♀ striped form, 17♀♀ black form), leg. MW; 24♂♂, 15♀♀ (6♀♀ striped form, 9♀♀ black form), leg. LK; 32♂♂, 25♀♀ (13♀♀ striped form, 12♀♀ black form), leg. WTS; 30 km NW of Biger [Бигэр] (45°50'N, 96°45'E), 1688 m a.s.l., 15 VIII 2015, body remains, leg. LK, WTS.

##### Remarks.

This is an endemic Mongolian species with its known distribution limited to a few localities in the northeastern part of Gobi-Altaj Aimag and southern Khovd Aimag. The imagines are active in July and August (Danilevsky 2007).

**Figure 13.**
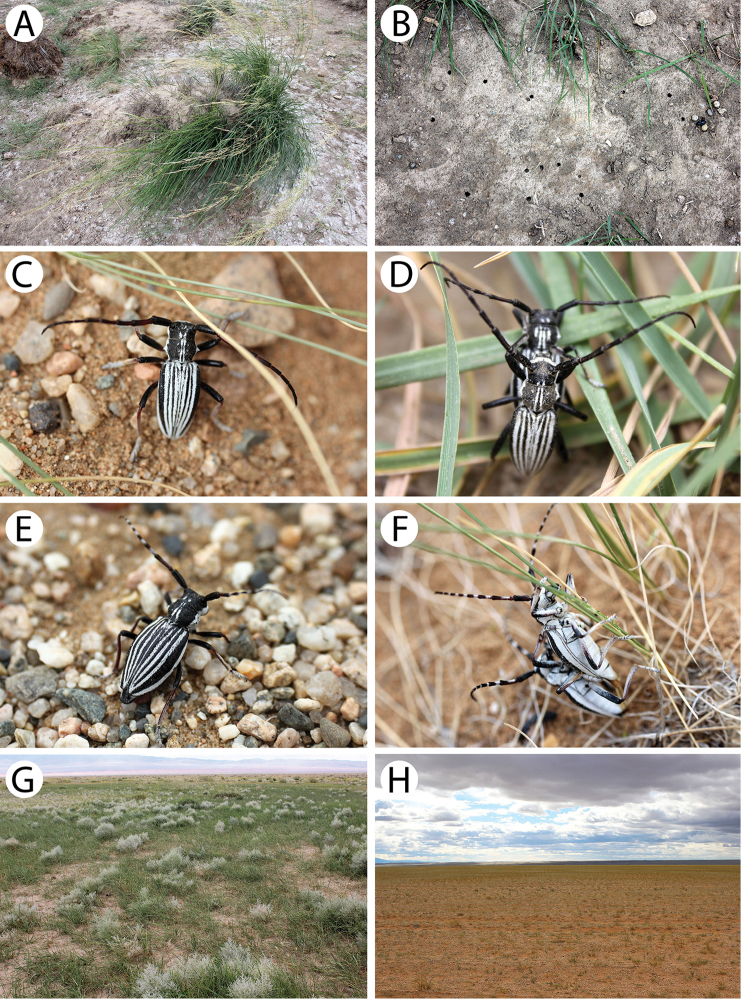
Field photos of imagines in nature and habitats of typical Mongolian cerambycid species: **A** tuft of needlegrass *Achnatherum splendens*, the possible host plant of larvae of *Eodorcadion consentaneum* **B** adults emergence holes of *E. consentaneum* in the sand **C** male of *Eodorcadion intermedium intermedium* (reddish form) **D** male of *Eodorcadion intermedium intermedium* (blackish form) **E** female of *E. intermedium intermedium* (reddish form) **F** pair of *E. intermedium intermedium in copula* **G** steppe in Baruunbayan-Ulaan environs, one of the habitat types of *E. intermedium intermedium* **H** semi-desert in Bogd environs, one of the habitat types of *E. intermedium intermedium*.

In the late evening (around 5–7 p.m.), despite the quite cold (15 °C) and cloudy weather with extremely gusty winds, we observed plenty (more than two hundred) of imagines that were rather active. Most of the observed specimens were mating (Fig. 12G); the ratio of males (Fig. 12D) and females was approximately equal. This was probably the climax of the appearance of this species. This was also confirmed by the very small number of dead individuals that have been found in the locality. Among the females, the black forms dominated (Fig. 12E) over the striped ones (Fig. 12F). The species inhabits the periphery of a drying lake with a sandy substrate and tufts of high grass growing out of ground elevations (Fig. 12H). The dominant plant species on the plot was *Achnatherum splendens* (Fig. 13A) and it is most probably the host plant of the larvae. Numerous emergence holes of the beetles were also observed in the sand (Fig. 13B).

Our own observations indicate that the species of the genus *Eodorcadion* seem to be more resistant to difficult weather conditions compared to other Dorcadionini. However, in the case of this taxon, although these conditions were extremely difficult, they apparently did not disturb the functioning and copulation of the beetles.

#### Eodorcadion intermedium intermedium

Taxon classificationAnimaliaColeopteraCerambycidae

(Jakovlev, 1889)

Figs 4F–I , 13C–F

##### Material examined.

Bayankhongor Aimag: 35 km SE of Bumbugur [Бөмбөгөр] (45°59'N, 99°50'E), 1598 m a.s.l., 16 VIII 2015, 3♂♂, 1♀ (including dead specimens), leg. MW; 1♀ (dead), leg. LK; 20 km NEE of Bogd [Богд] (45°17'N, 101°02'E), 1298 m a.s.l., 17 VIII 2015, 9♂♂, 3♀♀ (including dead specimens), leg. MW; 3♂♂, 2♀♀, leg. WTS; 7♂♂, 1♀ (including dead specimens; all with reddish antenna), leg. LK; 20 km SE of Bogd [Богд] (45°05'N, 101°08'E), 1263 m a.s.l., 18 VIII 2015, 1 dead imago, leg. LK; Övörkhangai Aimag: 10 km W of Baruunbayan-Ulaan [Баруунбаян-Улаан] (45°08'N, 101°14'E), 1264 m a.s.l., 18 VIII 2015, 2♂♂, leg. WTS; 5 km W of Baruunbayan-Ulaan [Баруунбаян-Улаан] (45°10'N, 101°17'E), 1266 m a.s.l., 18 VIII 2015, 11♂♂, 12♀♀, leg. MW; 4♂♂, 2♀♀ (specimens with black antenna (Fig. 13D)), leg. WTS; 4♂♂, 2♀♀ (most specimens with black antenna), leg. LK.

##### Remarks.

**Figure 14.**
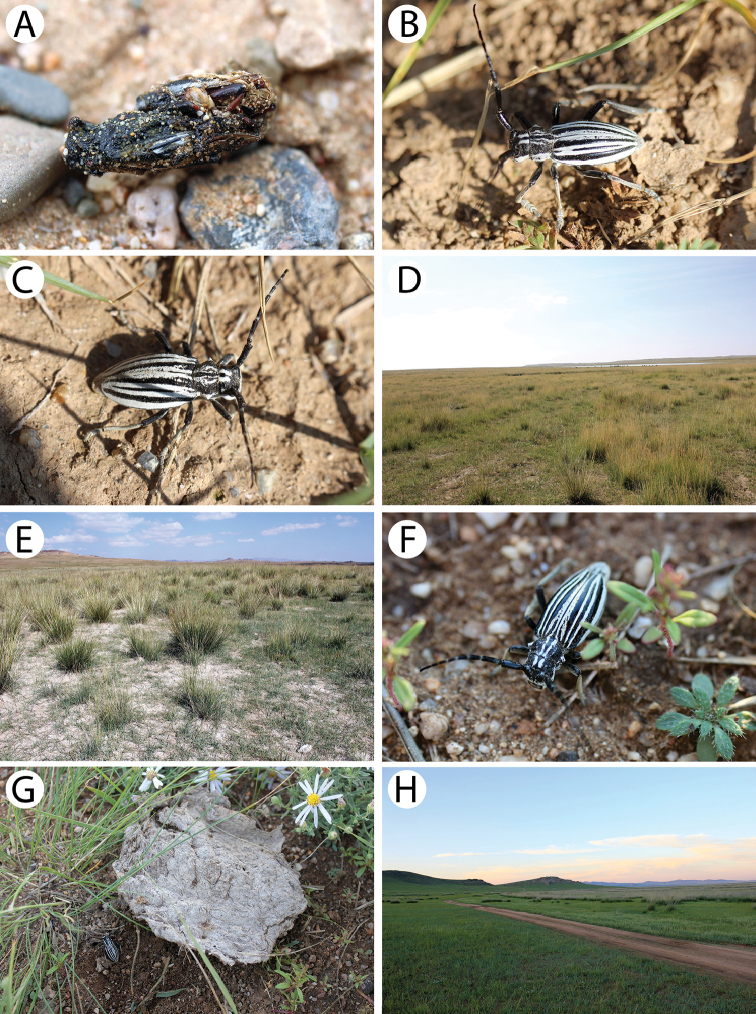
Field photos of imagines in nature and habitats of typical Mongolian cerambycid species: **A** remains of imago of *Eodorcadion intermedium intermedium* in a bird’s pellet **B** male of *Eodorcadion oryx* **C** female of *E. oryx* **D** periphery of a drying lake with a mix of high and low grass, the habitat of *E. oryx* and *Eodorcadion humerale impluviatum* **E** detailed view of the habitat of *E. oryx* and *E. humerale impluviatum* **F** female of *Eodorcadion exaratum argali* **G** female of *E. exaratum argali* under cow dung **H** pasture with low grass and herbs in Ulaanshiveet environs, the habitat of *E. exaratum argali*.

The species is widespread in the southern parts of Mongolia, where it is divided into two subspecies. The nominative subspecies is distributed in the western part of its range, where it occurs in many localities mainly in Bayankhongor and Gobi-Altaj Aimags. According to Danilevsky (2007), this taxon is characterised by a great deal of individual and geographical variability. We also observed elytral stripes – similar to *E. intermedium kozlovi* (Suvorov 1912) – and antennal colour variations, even in the case of specimens that were collected from the same locality. The species seems to have the ability to adapt to a wide range of ecological conditions; we observed it in various localities in high-grass enclaves, steppe and semi-desert habitats (Fig. 13G, H). The males (Fig. 13C) were primarily collected at different times of the day during rather cloudy (occasional shower) and cold (15-20 °C) weather; only a few pairs were copulating (Fig. 13F). Many specimens were already dead, which indicates the end of the appearance of this species. According to Danilevsky (2007), *E. intermedium* is ecologically associated with *Lasiagrostis*. At one of the localities, we found the remains of imago in a bird’s pellet (Fig. 14A).

#### Eodorcadion oryx

Taxon classificationAnimaliaColeopteraCerambycidae

(Jakovlev, 1895)

Figs 4B, C , 14B, C

##### Material examined.

Bayankhongor Aimag: 10 km S of Khairkhandulaan [Хайрхандулаан] (45°48'N, 101°59'E), 1748 m a.s.l., 18 VIII 2015, 1♂; 19 VIII 2015, 13♂♂, 2♀♀, leg. LK; 16♂♂, 6♀♀, leg. MW; 7♂♂, 2♀♀, leg. WTS.

##### Remarks.

*Eodorcadion oryx* is an endemic Mongolian species that is distributed in the south-western part of the country. It is a species that has very rarely been collected and there are records from only two known localities to date (Danilevsky 2007).

In the investigated locality, the species inhabits the periphery of a drying lake that has a mix of high and low grass (Fig. 14D, E). We collected most of the specimens before noon (10 a.m. – 12 p.m.) during sunny weather. Although the population was dominated by males (Fig. 14B), females (Fig. 14C) probably began to come out of hiding a little later. The day before, in the late evening, only lifeless imagines were observed, i.e., freshly dead female at the front of a rodent hole.

#### Eodorcadion exaratum argali

Taxon classificationAnimaliaColeopteraCerambycidae

(Jakovlev, 1889)

Figs 4D, E , 14F, G

##### Material examined.

Bulgan Aimag, 20 km N of Ulaanshiveet, (47°37'N, 103°51'E), 1108 m a.s.l.; 19 VIII 2015: 6♂♂, 3♀♀, leg. MW; 3♂♂, 4♀♀, leg. WTS; 1♂, 4♀♀, leg. LK.

##### Remarks.

This subspecies is distributed in the eastern part of Mongolia from the western boundary of Khentey Aimag to the Chinese border (Danilevsky 2007).

The adults are active at the turn of July and August. Danilevsky (2007) observed imagines of this species feeding on *Caragana* stems. This shrub has not been found in the presented locality.

In the late evening hours (about 8–9 p.m.), the specimens were collected during slightly windy weather from a habitat (pasture) that was mainly covered with low grass and herbs (Fig. 14H). In this locality, most of the beetles were hidden under rocks and cow dung (Fig. 14G).

#### Eodorcadion humerale impluviatum

Taxon classificationAnimaliaColeopteraCerambycidae

(Faldermann, 1833)

Figs 2D–G , 15A–D

##### Material examined.

Töv Aimag: 80 km NE of Ulaanbaatar [Улаанбаатар] (48°13'N, 107°43'E), 1778 m a.s.l., 31 VII 2015, 17♂♂, 7♀♀, leg. MW; 9♂♂, 4♀♀, leg. LK; 11♂♂, 1♀, leg. WTS (exclusively smaller and darker forms); Bayankhongor Aimag: 35 km SE of Bumbugur [Бөмбөгөр] (45°59'N, 99°50'E), 1598 m a.s.l., 16 VIII 2015, 9♂♂, 7♀♀, leg. MW; 4♂♂ (including 3 dead specimens), leg. WTS; 3♂♂, 1♀, leg. LK (exclusively yellow coloured forms); 33 km S of Nariinteel [Нарийнтээл]; (45°39'N, 101°22'E), 1626 m a.s.l., 17 VIII 2015, 1♂ (dead specimen), leg. MW; 1♀ (dead specimen), leg. LK; 10 km S of Khairkhandulaan [Хайрхандулаан] (45°48'N, 101°59'E), 1748 m a.s.l., 18–19 VIII 2015, 2♂♂, leg. LK; 1♂, leg. MW; 1♂, leg. WTS.

##### Remarks.

This widespread species, which contains three subspecies, is distributed in Russia from Transbaikalia to the Pacific Ocean in the Primorsky region, in the central and north-eastern parts of Mongolia and in north-eastern China. *Eodorcadion humerale humerale* is limited to the territory of Mongolia, where it has many known localities in the areas of Bayankhongor Aimag and Ulaanbaatar (Danilevsky 2007).

**Figure 15.**
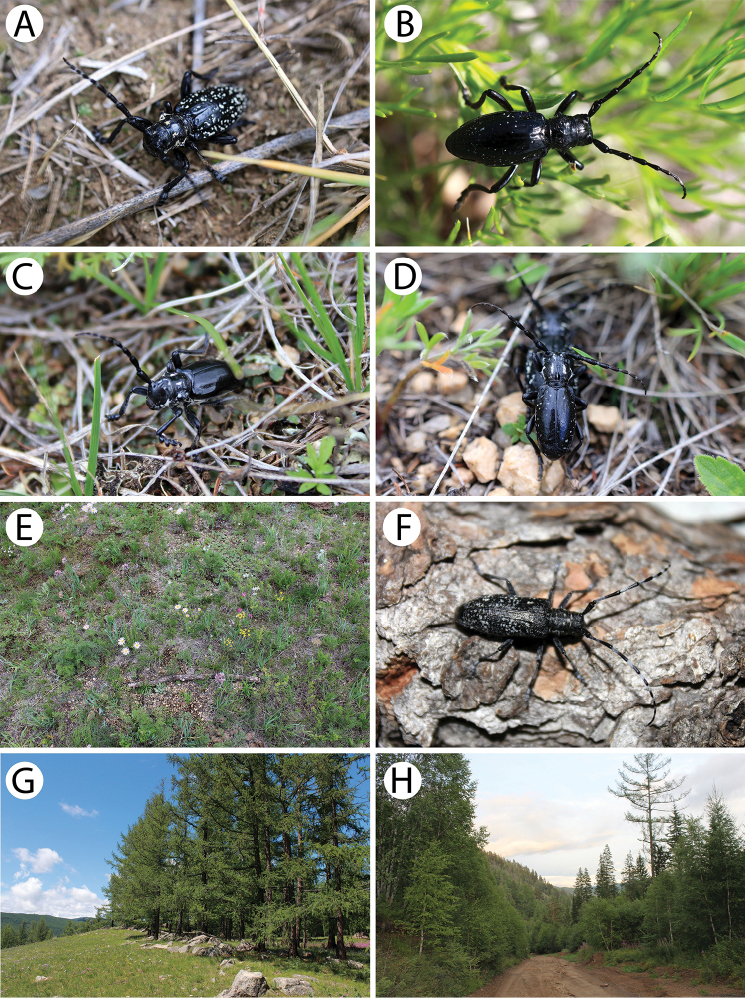
Field photos of imagines in nature and habitats of typical Mongolian cerambycid species: **A** male of *Eodorcadion humerale impluviatum* (typical form) **B** male of *E. humerale impluviatum* (Khentey Mountains) **C** male of *E. humerale impluviatum* (Khentey Mountains, entirely black elytra form) **D** pair of *E. humerale impluviatum in copula* (Khentey Mountains) **E** detailed view of the habitat of *E. humerale impluviatum* (Khentey Mountains) **F** female of *Monochamus impluviatus impluviatus* **G** larches in forest steppe, the habitat of *M. impluviatus impluviatus* **H** various species of coniferous trees and birches in dark taiga, the habitat of *Monochamus sartor urussovii*.

This taxon was observed sympatrically with other *Eodorcadion* species, i.e., *E. intermedium* and *E. oryx*, in steppe habitats (Fig. 14D) that had tufts of high grass (*inter alia Achnatherum splendens*). In addition to the typical form (Fig. 15A), that was collected in most of the presented localities, in the Khentey Mountains, we found a specific population characterised by a definitely smaller body size and by elytra covered with fewer white spots of hairs (sometimes almost completely black) (Fig. 15B–D). This population inhabits a xerothermic slope with a rich plant community on the edge of a larch woodlot in a forest-steppe habitat (Figs 10E, 15E). During rather cloudy weather before a storm, some specimens were still active and copulating (Fig. 15D) in the afternoon hours (about 1 p.m.). On the same plot, we also observed approximately ten specimens of *E. carinatum involvens* and one female of *Monochamus impluviatus impluviatus*. Due to the considerable dissimilarity in body size and type of biotope, this population requires further research and possibly represents a transitional form between two subspecies (*E. h. impluviatum* and *E. h. humerale*).

#### Monochamus impluviatus impluviatus

Taxon classificationAnimaliaColeopteraCerambycidae

(Motschulsky, 1859)

Figs 5D, E , 15F

##### Material examined.

Töv Aimag: 75 km NE of Ulaanbaatar (48°10'N, 107°55'E), 1589 m a.s.l., 30 VII 2015 (1 II 2016, ex cult), 1♂, 1♀, from *Larix sibirica*, leg. MW; 80 km NE of Ulaanbaatar (48°13'N, 107°43'E), 1778 m a.s.l., 31 VII 2015, 1♀, leg. WTS.

##### Remarks.

This is a Siberian species that is distributed from Ural to the Far East, including northern Mongolia, China, and North Korea (Sama 2002, Danilevsky 2017a). *Monochamus impluviatus* is a comparatively rare species that feeds exclusively on larch *Larix*. One generation takes two years to complete. The imagines are active from June to the first half of August. The species is sometimes found sympatrically with *Acanthocinus carinulatus* Gebler, 1833 and *Rhagium inquisitor* (Linnaeus, 1758) (Cherepanov 1990c).

Two specimens were also recorded from Bulgan Aimag by Heyrovský (1967a).

A single female (Fig. 15F) was found moving on the ground under a larch tree (Fig. 15G). One couple was additionally reared from a branch of a fallen *Larix sibirica* collected in forest steppe habitat (Fig. 7D). The same material was inhabited by larvae of *Clytus arietoides* and *Monochamus sutor*.

#### Monochamus sutor longulus

Taxon classificationAnimaliaColeopteraCerambycidae

(Pic, 1898)

Fig. 5F

##### Material examined.

Töv Aimag: 75 km NE of Ulaanbaatar (48°10'N, 107°55'E), 1589 m a.s.l., 30 VII 2015 (26 II 2016, ex cult), 1♀, from *Larix sibirica*, leg. MW.

##### Remarks.

*Monochamus sutor* is a boreal montane species that is widely distributed in Europe; in Asia, it is known from Georgia, Russia, Kazakhstan, and Mongolia. It is also an invasive species in North America (Danilevsky 2017a). This species is ecologically associated with various conifer trees. Its life cycle lasts from one to three years. The imagines are active from June to mid-September (Cherepanov 1990c, Kolk and Starzyk 1996).

*Monochamus sutor longulus* has a more eastern range compared to the nominative subspecies, and is distributed from East Siberia through northern Mongolia, China and North Korea to the Far East and Japan. It differs from the nominative form *inter alia* in its slightly more elongated elytra with glabrous and shining surface. According to Wallin et al. (2013), there is no difference in the male genitalia characters between the examined specimens of those two subspecies.

In Mongolia, this taxon was probably incorrectly identified in certain works (e.g., Heyrovský 1965, 1969, 1973a) and it was recorded as Monochamus sutor var. pellio (Germar, 1818), which is currently recognized as a synonym of the nominative subspecies.

One female was reared from a branch of a fallen tree of *Larix sibirica* collected in forest steppe habitat (Fig. 7D). The same material was inhabited by larvae of *Clytus arietoides* and *Monochamus impluviatus*.

#### Monochamus sartor urussovii

Taxon classificationAnimaliaColeopteraCerambycidae

(Fischer von Waldheim, 1805)

Fig. 5G

##### Material examined.

Selenge Aimag: 50 km NE of Zuunkharaa (49°05'N, 107°17'E), 930 m a.s.l., 03 VIII 2015, 1♂, leg. LK; several larvae, *Larix sibirica* leg. MW; Selenge Aimag: 35 km NE of Zuunkharaa (48°59'N, 106°55'E), 1399 m a.s.l., 05 VIII 2015, 1♂, leg. MW.

##### Remarks.

The taxonomic status of this species is uncertain. Cesari et al. (2005), Sláma (2006), and Wallin et al. (2013) considered *M. urussovii* to be a subspecies of *Monochamus sartor* (Fabricius, 1787). This taxon is widespread in Siberia and is distributed from Eastern Europe to the Far East and Japan (Danilevsky 2017a). Depending on the region, the larvae can develop in various conifers (mostly in *Abies* and *Picea*) and also sporadically on deciduous trees (Cherepanov 1990c, Wallin et al. 2013). In the Mongolian taiga, in addition to conifers, this species was found on birches (Müller et al. 2013). Its larval development usually takes two years. The imagines are active from the second half of May to the end of September (Cherepanov 1990c). Two single males were caught flying in both light and dark taiga (Fig. 15H).

#### Mesosa myops

Taxon classificationAnimaliaColeopteraCerambycidae

(Dalman, 1817)

Figs 5B, C , 16A

##### Material examined.

Selenge Aimag: 50 km NE of Zuunkharaa (49°05'N, 107°17'E), 930 m a.s.l., 02 VIII 2015, numerous larvae and pupae, 1♂, 1♀, *Betula platyphylla*, leg. LK, WTS, MW; (III 2016, ex larva), 1♂, leg. MW; (02–10 VIII 2015, ex pupa), 1♂, 5♀♀, leg. WTS; (05 VIII 2015, ex pupa), 2♂♂, 1♀, leg. LK.

##### Remarks.

This species is distributed from Eastern Europe (where it reaches eastern Poland) through Siberia, including northern Mongolia and China, to the Far East and Japan (Sama 2002, Danilevsky 2017a). It is listed in the Annexes of the European Habitats Directive (92/43/EEC), and therefore, it is strictly protected in the entire European Union. However, in the centre of its range (including Mongolia), *M. myops* is considered to be a common species and it is often numerously found in this region (e.g., Müller et al. 2013). This species is polyphagous on a large number of deciduous trees and shrubs. Its larval development usually takes two years. The pupation of the larvae takes place in the summer. After emerging from the pupae, the adult beetles emerge from their pupal cells from July to September; afterwards they probably overwinter in leaf litter. The imagines occur throughout entire warm season from May to September (Cherepanov 1990c).

**Figure 16.**
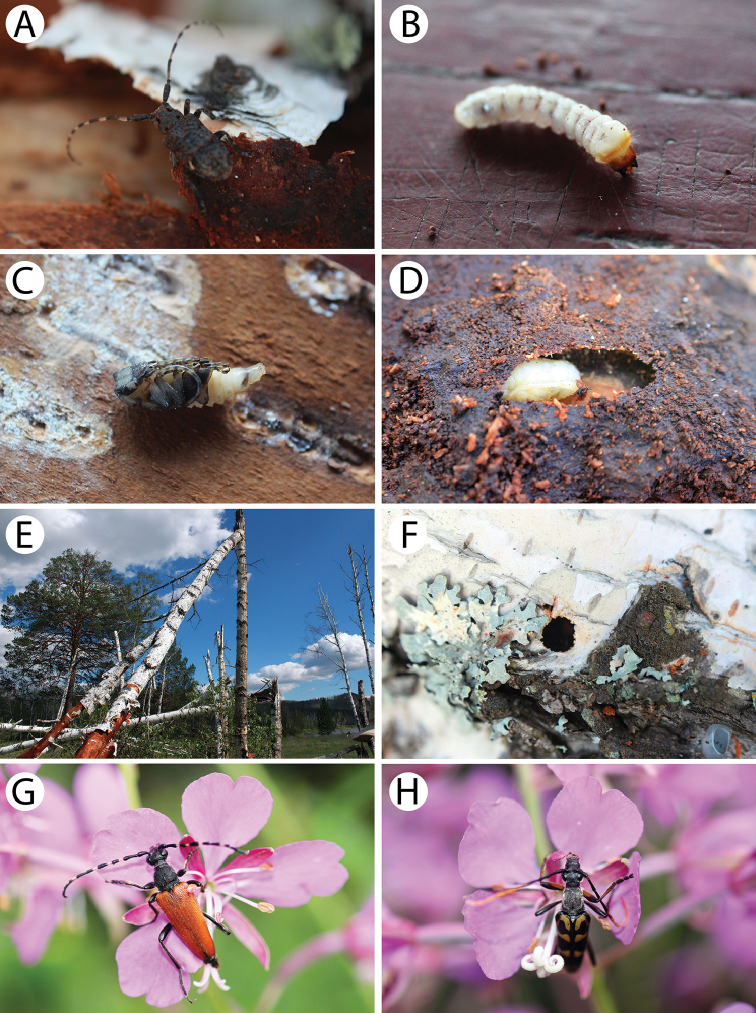
Field photos of imagines in nature, their immature stages and habitats of typical Mongolian cerambycid species: **A** newly emerged male of *Mesosa myops* **B** larva of *M. myops* **C** one of the last pupal instars of *M. myops* **D** pupa of *M. myops* in thick layer of cambium under the bark of broken birch trunk **E** broken trunk of the birch in light taiga, the microhabitat of *M. myops* **F** adults emergence holes of *M. myops* **G** *Stictoleptura variicornis* on *Chamaenerion angustifolium* **H** *Leptura annularis* on *Chamaenerion angustifolium*.

Several dozen larvae (Fig. 16B), some pupae (Fig. 16C, D) and newly emerged imagines (Fig. 16A) were observed under the bark (Fig. 9H) of rather thin broken *Betula platyphylla* (5–20 cm in diameter) (Fig. 16E) in the light taiga habitat (Fig. 7F) at the beginning of August. Several emergence holes of adults were also found on both branches and stems (Fig. 16F). The same material was additionally inhabited by larvae of *Xylotrechus hircus*, *Aegomorphus obscurior* and *Saperda scalaris*.

## Discussion

Many international expeditions (i.e., the USSR, Hungary, Poland, Germany) together with local specialists have intensively studied the Mongolian entomofauna in the second half of the 20^th^ century. The results of this research on different taxonomic groups of insects have been published in many different journals. Among those which were elaborated in the USSR, eleven volumes of scientific papers on Mongolian insect fauna (1972–1990) were published as part of the series “Insects of Mongolia”. The main emphasis was placed on two large orders of Insects: Coleoptera and Lepidoptera (Kovalenko et al. 2016).

The greatest contribution regarding Mongolian longhorn beetles has been made by Namhaidorzh (1972, 1974, 1976a,b, 1979, 1982) and Heyrovský (1964, 1965, 1967a,b, 1968, 1969, 1970, 1973a,b, 1975), the latter of whom elaborated the material collected by Zoltán Kaszab during his six expeditions conducted between 1963 and 1968, in the series entitled “Ergebnisse der Zoologischen Forschungen von Dr. Z. Kaszab in der Mongolei”. Additional data in this field was also published, *inter alia*, by Faldermann (1833), Jakovlev (1889), Janovsky (1974, 1977, 1980), Murzin (1977), Lindeman and Lyamtseva (1979), and Niisato (1994).

Despite all of these studies, the Mongolian cerambycid fauna is still not sufficiently recognised. This is evidenced by recently published descriptions of a new genus (*Rapuzziana* Danilevsky, 2006), several new species (*Pachytella churkini* Danilevsky, 2011; *Xylotrechus medvedevi* Danilevsky, 2009; *Eodorcadion savitskyi* Danilevsky, 2014; *E. gorbunovi* Danilevsky, 2004) and many subspecies (e.g., *E. rubrosuturale kerulenum* Danilevsky, 2007; *E. maurum australis* Danilevsky, 2014; *Cleroclytus semirufus savitsky* Lazarev, 2014). Moreover, some already known taxa might still be found here as new to the country, e.g., *Exocentrus stierlini* (Ganglbaur, 1883), which was recorded by Müller et al. (2013) from the Khan Khentey region.

In addition to taxonomic studies, the biology and ecology of Mongolian longhorn beetles (especially endemic species) should also be thoroughly investigated. For example, in this work, we report on the clearly important ecological role of the fireweed *Chamaenerion angustifolium* (= *Epilobium angustifolium*) (Onagraceae) in the life processes of many boreal anthophilous species, which has largely been neglected in the literature on the subject. This plant species, which is native throughout the temperate Northern Hemisphere, is very common in both light and dark Mongolian taiga. We observed ecological relationships between this plant and several cerambycid species: *Pachyta lamed*, *P. quadrimaculata*, *Gaurotes virginea*, *Stictoleptura variicornis* (Fig. 16G), *Anastrangalia sequensi*, *Lepturobosca virens*, *Leptura aethiops*, *L. annularis* (Fig. 16H), and *Stenurella melanura*. Numerous individuals of those species not only feed on *Chamaenerion angustifolium* but they also used the calyces of the flowers of this plant to protect themselves from the rain and to overnight in. Most of the above-mentioned cerambycids appeared to prefer this plant compared to other Asteraceae and Rosaceae occurring in these habitats.

Research on beetles, and on insects in general, in Mongolia is very important due to its pristine habitats. Consequently, some ecological patterns can still be studied here in comparatively untouched habitats. The Mongolian endemics are particularly vulnerable and unique. Moreover, since the territory of this country is located between Russia and China, it may constitute a transit zone for the establishment of some quarantine pests from southern Asia. Therefore, from the point of view of science, it is extremely important to preserve these more and more vulnerable habitats.

Mongolian ecosystems are under unprecedented pressures. The climate change occurring globally happens at a much greater rate in Mongolia than the global average (the mean annual temperature has increased 2.14 °C in the last 70 years, MNET 2009b, MEGD 2014). This warming trend, coupled with changes in the precipitation patterns (Goulden et al. 2016, Vandandorj et al. 2017), results in an overall drying tendency of ecosystems and the loss of surface waters. Another big challenge is the increase of livestock since it was privatised in 1992. In December 2016, Mongolia had 61.5 million heads of livestock, which was the highest number of free-ranging animals in the country (NSO 2016), thus causing overgrazing in many areas, especially near settled areas and water bodies. Even though the Mongolian ecosystems have been subject to pastoral livestock grazing, they have not been subjected to this level of livestock grazing pressure which is impacting the ecosystems simultaneously with the climate change. Both factors have been the main cause of land degradation in the country, even though simultaneously effects of which have been difficult to determine. However, the effects of grazing on insect diversity have been documented for certain groups of insects, e.g., moths (Enkhtur et al. 2017). According to some reports, close to 78 per cent of the Mongolian territory has been affected by land degradation (MEGD 2014). Although one could question the methodology of such an estimate, it is a clear sign of land degradation and desertification. Finally, another big factor in the local and regional-scale changes is the mining industry (MNET 2011). Over the last couple of decades, the Mongolian government has been encouraging foreign mining companies to invest and to start businesses in Mongolia (Farrington 2005). Various environmental issues have been raised as a consequence of mining, such as the development of, or lack thereof, linear infrastructures, elevated levels of dust and heavy metals pollution and threats to access to water resources.

## Supplementary Material

XML Treatment for Pachyta lamed

XML Treatment for Pachyta quadrimaculata

XML Treatment for Gaurotes virginea aemula

XML Treatment for Stictoleptura variicornis

XML Treatment for Anastrangalia sequensi

XML Treatment for Lepturobosca virens

XML Treatment for Pachytodes longipes

XML Treatment for Oedecnema gebleri

XML Treatment for Macroleptura thoracica

XML Treatment for Leptura aethiops

XML Treatment for Leptura annularis

XML Treatment for Lepturalia nigripes rufipennis

XML Treatment for Stenurella melanura

XML Treatment for Clytus arietoides

XML Treatment for Xylotrechus hircus

XML Treatment for Xylotrechus pantherinus

XML Treatment for Amarysius altajensis

XML Treatment for Anoplistes halodendri minutus

XML Treatment for Aegomorphus obscurior

XML Treatment for Saperda similis

XML Treatment for Saperda scalaris hieroglyphica

XML Treatment for Saperda alberti

XML Treatment for Agapanthia pilicornis pilicornis

XML Treatment for Eodorcadion carinatum involvens

XML Treatment for Eodorcadion maurum australe

XML Treatment for Eodorcadion dorcas dorcas

XML Treatment for Eodorcadion dorcas scabrosum

XML Treatment for Eodorcadion consentaneum

XML Treatment for Eodorcadion intermedium intermedium

XML Treatment for Eodorcadion oryx

XML Treatment for Eodorcadion exaratum argali

XML Treatment for Eodorcadion humerale impluviatum

XML Treatment for Monochamus impluviatus impluviatus

XML Treatment for Monochamus sutor longulus

XML Treatment for Monochamus sartor urussovii

XML Treatment for Mesosa myops
